# Segment-Specific Neuronal Subtype Specification by the Integration of Anteroposterior and Temporal Cues

**DOI:** 10.1371/journal.pbio.1000368

**Published:** 2010-05-11

**Authors:** Daniel Karlsson, Magnus Baumgardt, Stefan Thor

**Affiliations:** Department of Clinical and Experimental Medicine, Linkoping University, Linkoping, Sweden; University of Cambridge, United Kingdom

## Abstract

The generation of distinct neuronal subtypes at different axial levels relies upon both anteroposterior and temporal cues. However, the integration between these cues is poorly understood. In the *Drosophila* central nervous system, the segmentally repeated neuroblast 5–6 generates a unique group of neurons, the Apterous (Ap) cluster, only in thoracic segments. Recent studies have identified elaborate genetic pathways acting to control the generation of these neurons. These insights, combined with novel markers, provide a unique opportunity for addressing how anteroposterior and temporal cues are integrated to generate segment-specific neuronal subtypes. We find that Pbx/Meis, Hox, and temporal genes act in three different ways. Posteriorly, Pbx/Meis and posterior Hox genes block lineage progression within an early temporal window, by triggering cell cycle exit. Because Ap neurons are generated late in the thoracic 5–6 lineage, this prevents generation of Ap cluster cells in the abdomen. Thoracically, Pbx/Meis and anterior Hox genes integrate with late temporal genes to specify Ap clusters, via activation of a specific feed-forward loop. In brain segments, “Ap cluster cells” are present but lack both proper Hox and temporal coding. Only by simultaneously altering Hox and temporal gene activity in all segments can Ap clusters be generated throughout the neuroaxis. This study provides the first detailed analysis, to our knowledge, of an identified neuroblast lineage along the entire neuroaxis, and confirms the concept that lineal homologs of truncal neuroblasts exist throughout the developing brain. We furthermore provide the first insight into how Hox/Pbx/Meis anteroposterior and temporal cues are integrated within a defined lineage, to specify unique neuronal identities only in thoracic segments. This study reveals a surprisingly restricted, yet multifaceted, function of both anteroposterior and temporal cues with respect to lineage control and cell fate specification.

## Abstract 2

To address the question of how neuronal diversity is achieved throughout the CNS, this study provides evidence of modulation of neural progenitor cell “output” along the body axis by integration of local anteroposterior and temporal cues.

## Author Summary

An animal's nervous system contains a wide variety of neuronal subtypes generated from neural progenitor (“stem”) cells, which generate different types of neurons at different axial positions and time points. Hence, the generation and specification of unique neuronal subtypes is dependent upon the integration of both spatial and temporal cues within distinct stem cells. The nature of this integration is poorly understood. We have addressed this issue in the *Drosophila* neuroblast 5–6 lineage. This stem cell is generated in all 18 segments of the central nervous system, stretching from the brain down to the abdomen of the fly, but a larger lineage containing a well-defined set of cells—the Apterous (Ap) cluster—is generated only in thoracic segments. We show that segment-specific generation of the Ap cluster neurons is achieved by the integration of the anteroposterior and temporal cues in several different ways. Generation of the Ap neurons in abdominal segments is prevented by anteroposterior cues stopping the cell cycle in the stem cell at an early stage. In brain segments, late-born neurons are generated, but are differently specified due to the presence of different anteroposterior and temporal cues. Finally, in thoracic segments, the temporal and spatial cues integrate on a highly limited set of target genes to specify the Ap cluster neurons.

## Introduction

The generation of distinct neuronal cell types at different axial levels represents a crucial feature of nervous system development. This segment-specific neuronal subtype specification relies upon both anteroposterior and temporal cues, and significant progress has been made in understanding each of these two processes. Along the anteroposterior axis, a number of studies have revealed that the Hox homeotic genes play key roles, acting in several different ways to control segment-specific nervous system development (reviewed in [1]–[6]). In particular, studies of mammalian motoneuron development have revealed critical input from Hox genes, acting in surprisingly restricted ways to specify unique motoneuron identities [7]–[9]. Hox genes often act in tight interplay with Hox cofactors of the Pbx and Meis families [10],[11], and although less studied in the nervous system, these factors have also been found to play important roles during segment-specific cell fate determination [9]. However, our understanding of Hox/Pbx/Meis function in the nervous system is still rudimentary, in particular with respect to how these cues are integrated with lineage progression and with respect to their specific targets in the different settings. Along the temporal “axis” studies have revealed that neural progenitor cells undergo stereotypic temporal transitions in competence, which result in the generation of distinct cell types at different time points (reviewed in [12]). In *Drosophila*, a well-defined cascade of transcription factors, the temporal gene cascade of *hunchback-Kruppel-Pdm-castor-grainyhead*, is expressed in sequential fashion by most central nervous system (CNS) progenitors (neuroblasts), and control distinct “competence windows” in neuroblasts (reviewed in [13]). Despite progress with respect to anteroposterior control of nervous system development on the one hand, and to temporal changes in neuroblasts on the other, little is known regarding how these two fundamental developmental axes are integrated to establish distinct neuronal cell types at different axial levels.

**Figure 1:**
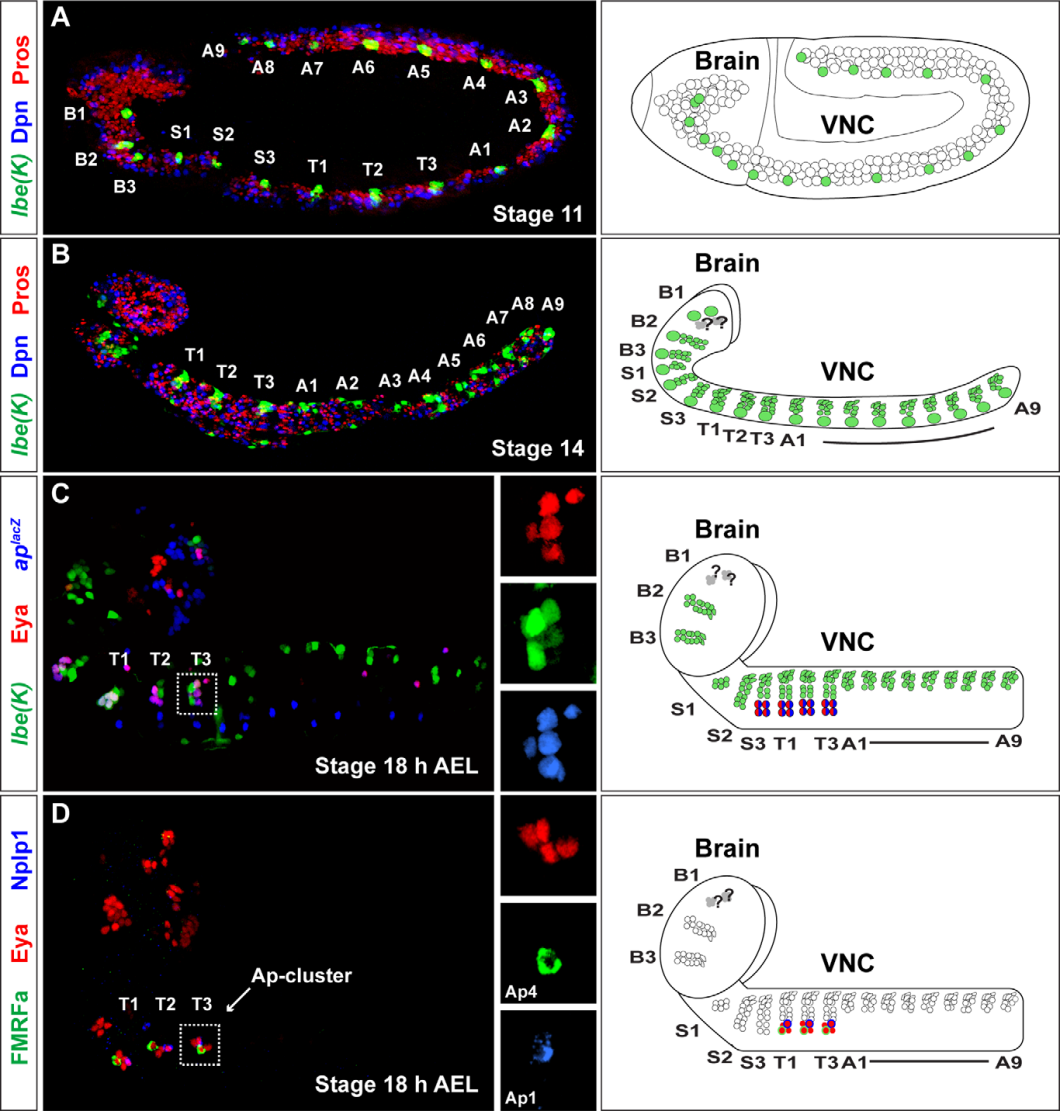
The Ap cluster is generated by thoracic neuroblast 5–6. The transgenic reporters *lbe(K)-lacZ* and *lbe(K)-Gal4* allow for visualization of the NB 5–6 lineage throughout the developing *Drosophila* CNS. (A) Expression of *lbe(K)-lacZ* at embryonic stage 11 in the NB 5–6 lineage in brain (B1–B3), subesophageal (S1–S3), thoracic (T1–T3), and abdominal segments (A1–A9). (B) At stage 14, the difference in size between NB 5-6A and NB 5-6T is becoming evident. To visualize the outline of the *Drosophila* CNS, the Deadpan and Prospero markers was used. (C) At stage 18 h AEL, coexpression of Eya, *ap^lacZ^*, and *lbe(K)-Gal4*, reveal that the four Ap cluster neurons (Ap1–4) are generated within the NB 5-6T lineage. Boxes to the right depict a T3 Ap cluster (dashed box in left panel), with the expression of Eya, *ap^lacZ^*, and *lbe(K)-Gal4* in separate panels. (D) Within the Ap cluster, the Ap1 and Ap4 neurons can be identified by the expression of the two neuropeptides Nplp1 (Ap1) and FMRFa (Ap4). Boxes to the right depict a T3 Ap cluster (dashed box in left panel), with the expression of Eya, Nplp1, and proFMRFa in separate panels. (A–D) are composed from multiple images. Genotypes: (A) *lbe(K)-lacZ*. (B) *lbe(K)-Gal4*, *UAS-nmEGFP/+*; *lbe(K)-Gal4*, *UAS-GFP/+*. (C) *ap^lacZ^/+*; *lbe(K)-Gal4/UAS-nmEGFP/+*. (D) *w^1118^*.

The developing *Drosophila* CNS is generated from a stereotyped set of some 1,000 neuroblasts (reviewed in [14]). They are organized into 18 segments: three brain segments (B1–B3), three subesophageal segments (S1–S3), three thoracic segments (T1–T3), and nine abdominal segments (A1–A9) (Figure 1A and 1B). These segments are typically referred to as the brain (B1–B3 through S1–S3) and the ventral nerve cord (VNC; T1–T3 through A1–A9) (Figure 1B). Neuroblasts undergo series of asymmetric cell divisions, “budding” off secondary progenitor cells denoted *ganglion mother cells* (GMCs), that in turn typically divide one final time to generate neurons and/or glia [15]–[18]. Each neuroblast has a unique and stereotypic identity, as revealed by the size of its lineage—ranging from two to 40—and by the types of neurons and glia generated [19]–[21]. Each thoracic and abdominal hemisegment contains 30 neuroblasts that delaminate from the ectoderm in seven distinct rows [22]. In each of the six thoracic hemisegments, the lateral-most thoracic row 5 neuroblast, NB 5-6T, generates a unique lateral cluster of four neurons—the Ap cluster—that specifically expresses the LIM-HD transcription factor Apterous (Ap) and the Eyes absent (Eya) cofactor (Figure 1A–1D) [23],[24]. Two of the Ap cluster neurons can be further identified by the specific expression of two neuropeptides—FMRFamide (FMRFa) and Nplp1 (Figure 1D) [25]–[27]—and the four Ap cluster neurons thus represent at least three distinct cell types: Ap1/Nplp1, Ap2/3 (ipsilaterally projecting interneurons), and Ap4/FMRFa (Figure 1D).

Studies have identified several genes acting to ensure proper Ap cluster specification and to activate the cell-specific expression of Nplp1 and FMRFa [23]–[34]. Moreover, to better understand the genetic mechanisms of Ap cluster specification, we recently resolved the entire NB 5-6T lineage, finding that Ap neurons are born at the end of this large lineage. We furthermore identified the temporal transitions that control generation of the three distinct Ap cluster neuronal cell types at the end of this lineage [35]. These studies revealed critical input from the two late temporal genes *castor* (*cas*) and *grainyhead* (*grh*). *cas* plays multiple roles to specify Ap neurons, one of which is to trigger a critical feed-forward loop involving the COE/Ebf family member *collier/knot* (*col*) [25]. In contrast, *grh* acts selectively to specify the Ap4/FMRFa neuron. Several combined elements presented us with a unique opportunity for addressing how an identifiable neural lineage is modified along the entire anteroposterior axis to generate segment-specific cell types, including; 1) the development of an NB 5–6–specific reporter and *Gal4* “driver” 2) the characterization of the NB 5-6T lineage, 3) the identification of a unique thoracic-specific group of cells generated by this lineage (the Ap cluster), 4) the highly restricted expression of the FMRFa and Nplp1 neuropeptides within two of the four Ap cluster neurons, and 5) the elucidation of an elaborate progenitor and postmitotic genetic pathway specifying the Ap cluster neurons.

We find that Ap cluster neurons exclusively appear in thoracic segments as a result of several distinct mechanisms, acting at the different axial levels. In the abdomen, the Hox genes of the bithorax complex (Bx-C)—*Ultrabithorax* (*Ubx*), *abdominal-A* (*abd-A*), and *Abdominal-B* (*Abd-B*)—act with the Pbx/Meis Hox cofactors encoded by *homothorax* (*hth*) and *extradenticle* (*exd*) genes, to terminate progression of the NB 5-6A lineage, via neuroblast cell cycle exit. This occurs within an early (Pdm) temporal window, thereby preventing the actual generation of Ap cluster neurons, as well as the progression into late temporal windows specified by *cas* and *grh*. In the thorax, the thoracic Hox gene *Antennapedia* (*Antp*) acts with *hth* and *exd* to specify Ap cluster neurons within NB 5-6T. Of the many possible ways in which Pbx/Meis and Hox input could control this event, we find that *Antp*, *hth*, and *exd* integrate with the temporal gene *cas* to specifically activate *col* and the *col*-mediated critical feed-forward loop. Intriguingly, we find that the actual levels of Hth expression acts in an instructive manner, acting at low levels to trigger neuroblast cell cycle exit in NB 5-6A, and at high levels to trigger *col* expression in NB 5-6T. In more anterior segments, equivalents of “Ap cluster cells” are generated, but fail to differentiate into Ap cluster neurons, not only due to the absence of *Antp* expression, but also due to absent or low-level expression of the temporal factor Grh, which is critical for specifying the Ap4/FMRFa cell fate. Co-misexpression of *Antp* with *grh* specifies Ap cluster neurons, with expression of the neuropeptides Nplp1 and FMRFa in anterior brain segments. By co-misexpressing *Antp* and *grh* in a Bx-C triple mutant background (*Ubx*, *abd-A*, *Abd-B*), a “thoracic CNS” is generated with Ap clusters emerging throughout the neuroaxis. In summary, the dynamic and restricted expression of Hox, Pbx/Meis, and temporal genes, coupled with their unique functions, act to modify an equivalent CNS lineage along the neuroaxis by three different mechanisms: 1) abdominal lineage size control, 2) thoracic integration upon a specific feed-forward loop, and 3) the anterior absence of proper Hox and temporal expression.

## Results

The NB 5–6 lineage was previously identified in both thoracic and abdominal segments [19],[20], and in addition, an equivalent lineage has been identified in the three brain segments [14],[36]–[38]. However, Ap clusters are exclusively generated by the six thoracic NB 5–6 lineages, but why? To follow the progression of the NB 5–6 lineage in the different CNS segments in more detail, we utilized the NB 5–6–specific transgenic markers *lbe(K)-lacZ* [39] and *lbe(K)-Gal4* [35]. NB 5–6 delaminates from the ectoderm at stage 9 [40], and we can observe reporter gene expression starting from stage 11 (Figure 1A). A single NB 5–6 is generated in each hemisegment of the developing CNS, with the exception of the first brain segment (B1) which contains two NB 5–6 equivalents (Figure 1; unpublished data) [14]. The early steps of NB 5–6 progression is similar in all segments, with the exception of the two 5–6 NBs in the B1 segment, where expression of GFP is not as robust and the lineage is difficult to follow throughout development (Figure 1A–1C).

### Expression of Hox and Pbx/Meis Factors within the NB 5–6 Lineage

In *Drosophila*, the control of segment identity is in part controlled by the homeotic (Hox) genes and the Pbx/Meis Hox cofactors, encoded by the *homothorax* (*hth*) and *extradenticle* (*exd*) genes [11],[41]. Mutations in these genes strongly affect both the abdominal and thoracic NB 5–6 lineage (see below). We thus mapped the expression of the relevant Hox factors; Antennapedia (Antp), the bithorax Hox complex (Bx-C) factors, Ultrabithorax (Ubx), Abdominal-A (Abd-A), and Abdominal-B (Abd-B), as well as Hth and Exd, in the NB 5–6 lineage (Figure S1 and S2).

**Figure 2:**
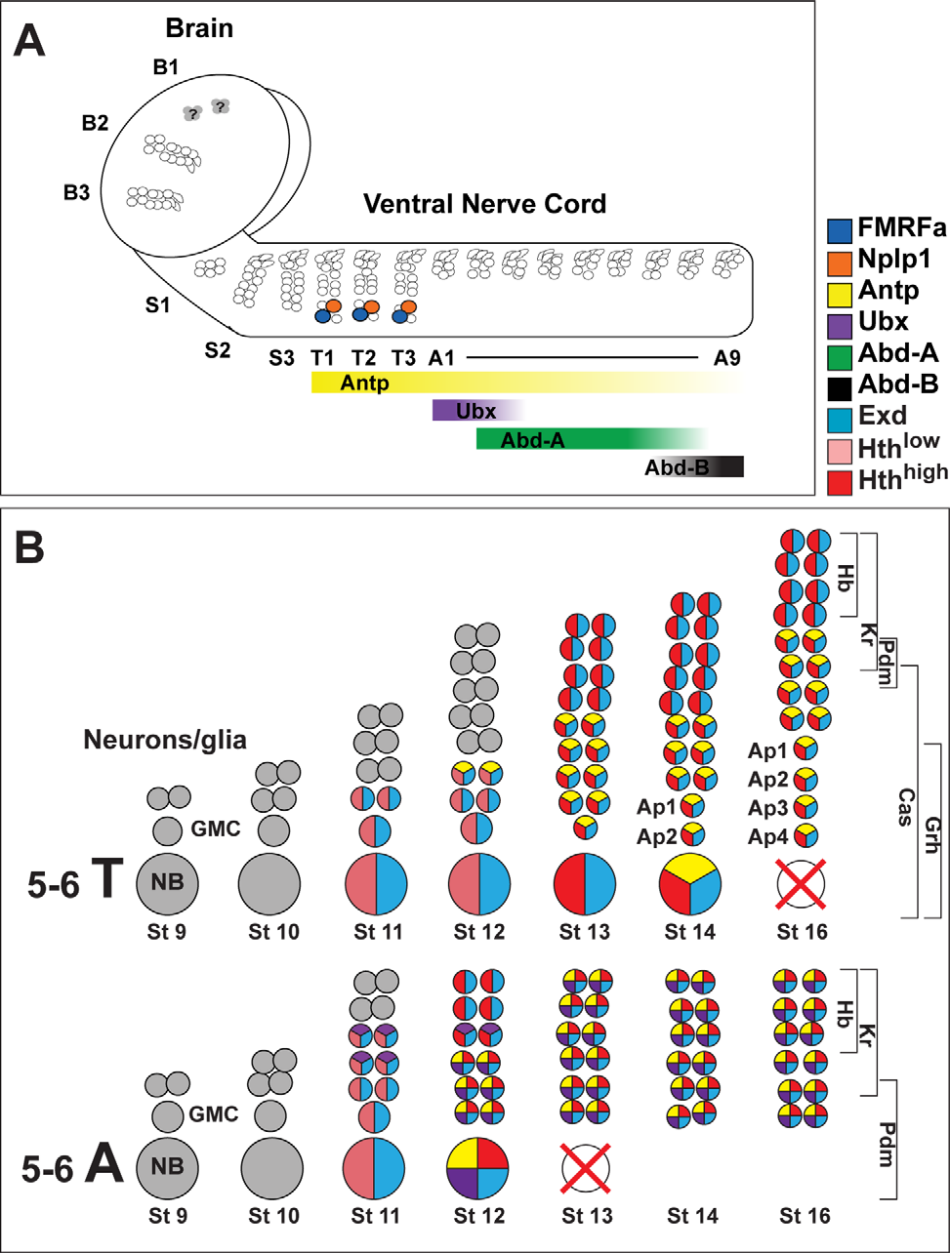
Expression of Hox, Pbx/Meis, and temporal factors within neuroblast lineage 5–6. (A) Cartoon depicting the NB 5–6 lineage in the 18 embryonic *Drosophila* CNS segments, with the anteroposterior limits of expression of Hox factors. Although the NB 5–6 lineage is present in all segments, the Ap clusters only appear in thoracic segments. (B) Cartoon depicting the NB 5-6A and NB 5-6T lineage, with expression of Hox, Pbx/Meis, and temporal factors outlined. During stage 9 and 10, only expression of early temporal genes is evident. At stage 11, expression of Hth and Exd commences, followed by Antp and Ubx at stage 12 (for simplicity, expression of Abd-A and Abd-B is not depicted). However, Ubx is not expressed in NB 5-6T at any point. Both NB 5-6A and 5-6T display the typical temporal gene progression of Hb-Kr-Pdm, but only NB 5-6T continues dividing and progressing into the Cas/Grh window. Previous studies reveal a striking drop in Cas expression in the neuroblast at stage 13, after which it commences again. After exiting the cell cycle at stage 12 (NB 5-6A) and 15 (NB 5-6T), both neuroblasts undergo apoptosis.

**Figure 3:**
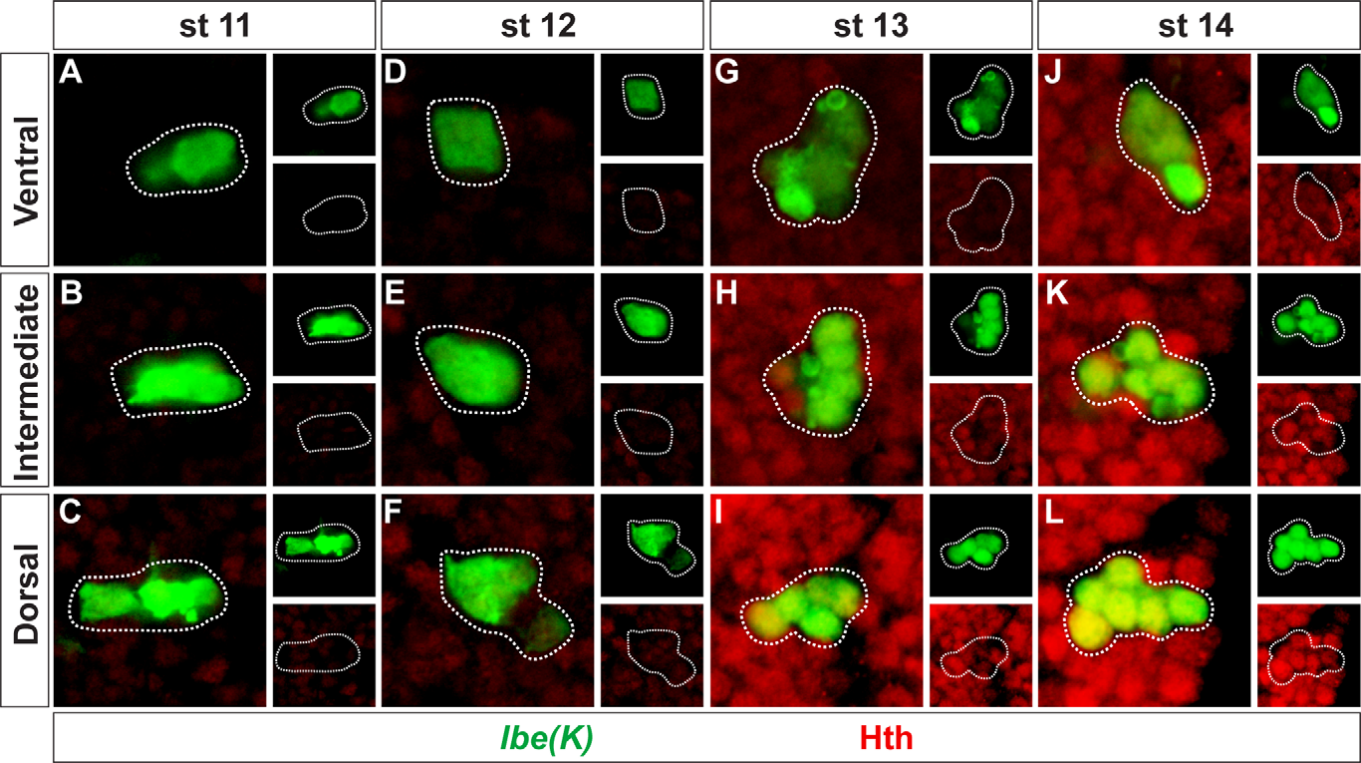
Homothorax levels rise sharply at stage 13. (A–L) T2 hemisegments of stage (st) 11 (A–C), 12 (D–F), 13 (G–I), and 14 (J–L) embryos, showing the expression of Hth within the NB 5-6T lineage. Small boxes show the expression of Hth and *lbe(K)-Gal4* in separate panels. All images were from embryos processed on the same slide, and scanned using identical confocal settings. There is a sharp increase in Hth levels between stage 12 (D–F) and 13 (G–I). Genotype: *lbe(K)-Gal4*, *UAS-nmEGFP*.

We find that expression of Hth and Exd commences in NB 5–6 at stage 11 in abdominal and thoracic lineages, is found in all cells within the lineages at stage 13, and is maintained throughout the lineages during subsequent stages (Figure S1; Figure 2B). Both genes are also expressed by more anterior NB 5–6 lineages (unpublished data). Hth is expressed at low levels initially, but increases rapidly at stage 13, in thoracic and anterior segments in general [42],[43] (unpublished data), as well as in thoracic and more anterior NB 5–6 lineages specifically (Figure 3). Antp is expressed in a gradient in the VNC, high anterior and low posterior, with the anterior limit in T1 (Figure S2A–S2C) [44]. In both NB 5-6A and NB 5-6T, Antp expression commences at stage 12, and is maintained in all cells born after this stage. Ubx expression commences within NB 5-6A at stage 11, and is subsequently expressed in earlier-born cells in this lineage in segments A1 to A7. Abd-A and Abd-B are expressed similarly to Ubx, with Abd-A in segments A2 to A9, and Abd-B in segments A5 to A9 (Figures S1 and S2; Figure 2A and 2B). Thus, Bx-C gene expression fits with a potentially suppressive role on Ap cluster formation, *Antp* expression with a potentially positive role, and *hth*/*exd* expression with dual roles.

### Absence of Ap Clusters in the Abdomen Results from a Truncated 5–6 Lineage

**Figure 4:**
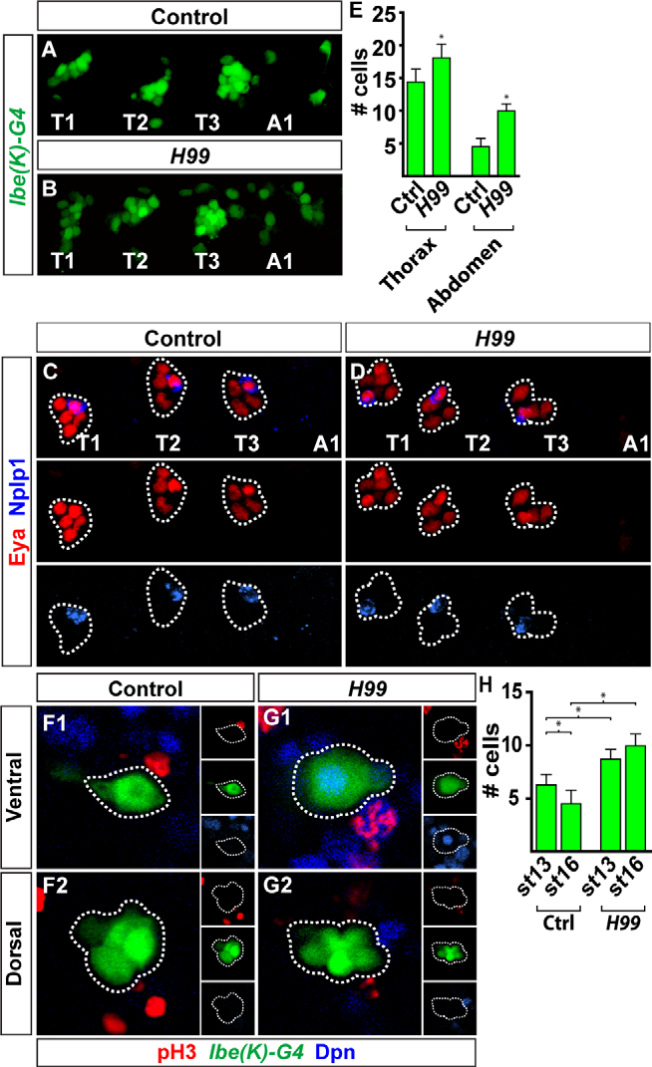
Genetically blocking cell death in the abdominal NB 5–6 lineage does not result in appearance of Ap cluster neurons. (A and B) Expression of *lbe(K)-Gal4* at stage 16, in control (A) and *H99* (B), reveals a clear difference in both the NB 5–6 thoracic and abdominal lineage size (side view, T1–A1). (C and D) Expression of Eya and Nplp1 at stage 18 h AEL, in control (C) and *H99* mutants (D) (side view, T1–A1). There is no evidence of ectopic Ap clusters in abdominal segments. (E) Quantifying NB 5–6 lineage cells in the thoracic versus the abdominal area, in control and *H99*, reveals that additional cells appear in both lineages when apoptosis is blocked. (F and G) Stage 13, ventral and dorsal confocal stacks of NB 5-6A visualized using *lbe(K)-Gal4*. Small boxes show the expression of pH3, Dpn, and *lbe(K)-Gal4* in separate panels. In contrast to control (F1 and F2), *H99* (G1 and G2) mutants reveal expression of Dpn in the NB. In contrast, no phospohistone-3 (pH3) staining is observed in either genotype beyond stage 13. (H) Quantifying NB 5-6A lineage cells at stage 13 in control and *H99* mutants reveals no significant increase in cell number in *H99* past stage 13. Data for stage 16 were copied from bar graph (E) for easy comparison. (E and H) Asterisks denote significant difference (*p*<0.01; Student two-tailed test). Genotypes: (A and F) *lbe(K)-Gal4,UAS-nmEGFP*. (B and G) *lbe(K)-Gal4,UAS-nmEGFP;H99*. (C) *w^1118^*. (D) *H99*.

**Figure 5:**
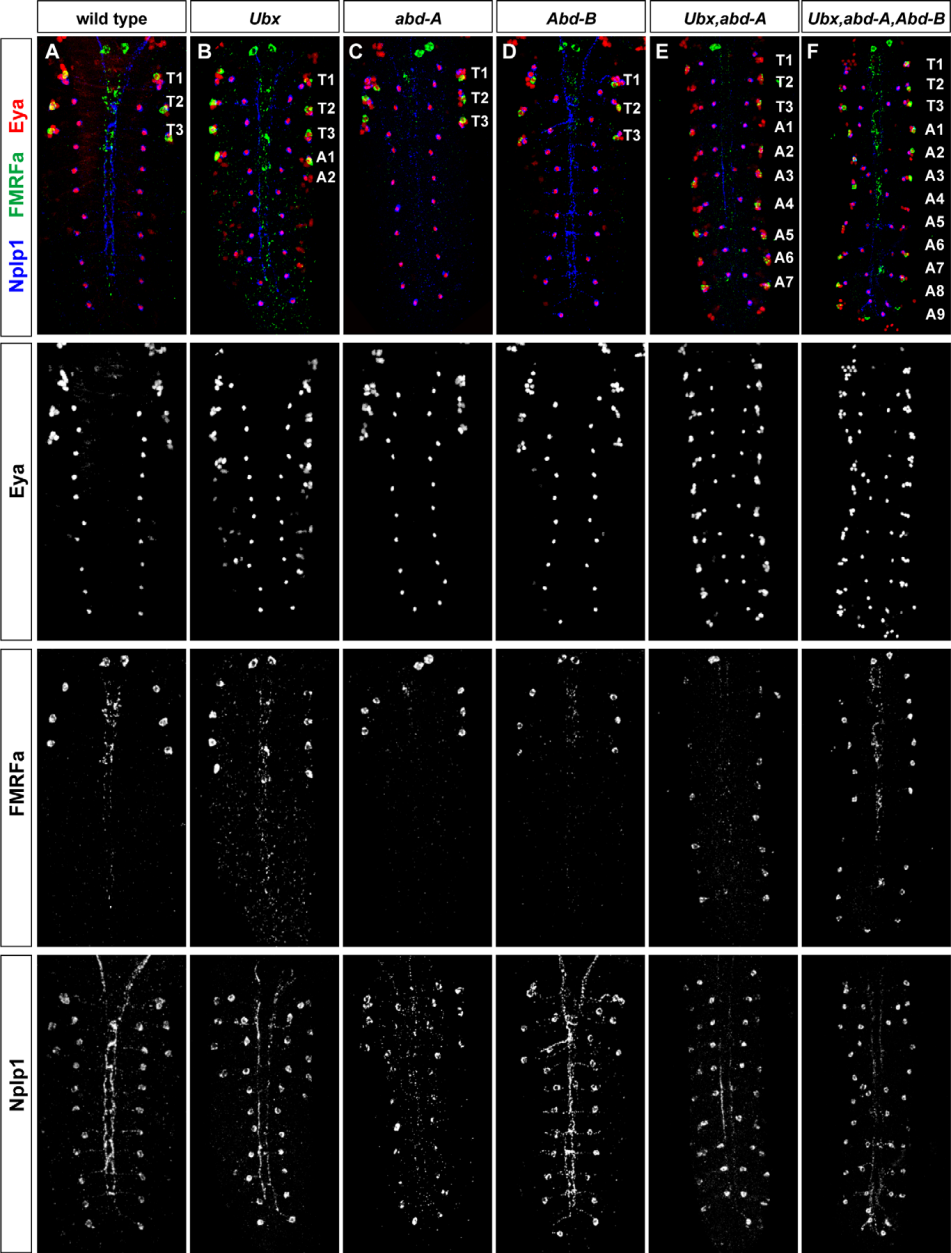
Homeotic transformations of the abdominal NB 5–6 lineage. (A–F) Analysis of Ap clusters, defined by expression of Eya, FMRFa, and Nplp1 in Bx-C single, double, and triple mutants, reveals the appearance of Ap clusters in abdominal segments. Due to genetic redundancy, in *Ubx* mutants (B), only A1 and partly A2 are transformed; in *abd-A* (C) and *Abd-B* (D), no segment is transformed; in *Ubx*, *abd-A* double mutants (E), A1–A8 are transformed, whereas in the triple mutant, all abdominal segments (A1–A9) are transformed (F). Stage 18 h AEL embryos. Genotypes: (A) *w^1118^*. (B) *Ubx^1^/Ubx^9.22^*. (C) *abd-A^MX1^*. (D) *Abd-B^M1^/Abd-B^M2^*. (E) *Ubx^109^*. (F) *Dp(3;1)P68*; *ss^1^ Ubx^1^ abd-A^D24^ Abd-B^D18^*.

We recently mapped the complete outline of the NB 5-6T lineage [35]. These studies revealed that the four Ap cluster neurons are the last-born cells within the NB 5-6T lineage, and that they are born within a Cas/Grh late temporal window (Figure 2B). We conducted a similar analysis of the NB 5-6A lineage (Figure S3). We find that NB 5-6A stops dividing at stage 12, within an earlier temporal window specified by Pdm, and thus ends up generating a smaller lineage when compared to the NB 5-6T (Figure 2B). These findings are in line with previous studies of the 5–6 lineage [19],[20]. As anticipated from these findings, there is no expression evident of the critical Ap cluster determinant Col (see below). We find apoptosis of four to five cells within the NB 5-6A lineage, but are unable to identify cleaved Caspase 3 staining unequivocally in the neuroblast (Figure S3L and S3M). Thus, the truncation of the NB 5-6A lineage could either result from an earlier cell cycle exit in the neuroblast, or from neuroblast apoptosis. To distinguish between these two possibilities, we analyzed NB 5-6A lineage progression in the *H99* deletion, a mutation that removes the three critical RHG-domain cell death genes *reaper*, *head involution defective*, and *grim*, and is well established not to display any embryonic apoptosis [45]. In *H99* mutants, Ap clusters do not appear in abdominal segments (Figure 4C and 4D). As anticipated from the apoptosis of four to five cells within the wild-type NB 5-6A lineage (Figure S3L and S3M), we find that four to five additional cells are present in *H99* (Figures 4A, 4B, and 5E). However, we do not find evidence of additional rounds of mitosis past stage 13 (Figure S3F and S3G; *n* = 12 hemisegments). In addition, the four to five additional cells observed in *H99* are observed already at stage 13 (Figure 4H). In the wild type, the neuroblast cannot be identified using Dpn (*n* = 14 hemisegments), but in contrast in *H99*, we are able to identify a ventral Dpn-positive cell (Figure 4F and 4G; nine out of 11 hemisegments).

These results demonstrate that NB 5-6A generates a truncated lineage, when compared to NB 5-6T, not due to apoptosis of the neuroblast, but rather due to an earlier cell cycle exit, within the Pdm window, followed thereafter by apoptosis. Thus, the lack of Ap clusters in abdominal segments represents the logical outcome of a truncated NB 5–6 lineage, since it never generates Ap cluster cells and never progresses into the late competence window specified by the Cas and Grh temporal factors, both of which are critical for Ap cluster specification.

### Posterior Hox Genes Act to Truncate the Abdominal NB 5–6 Lineage

**Figure 6:**
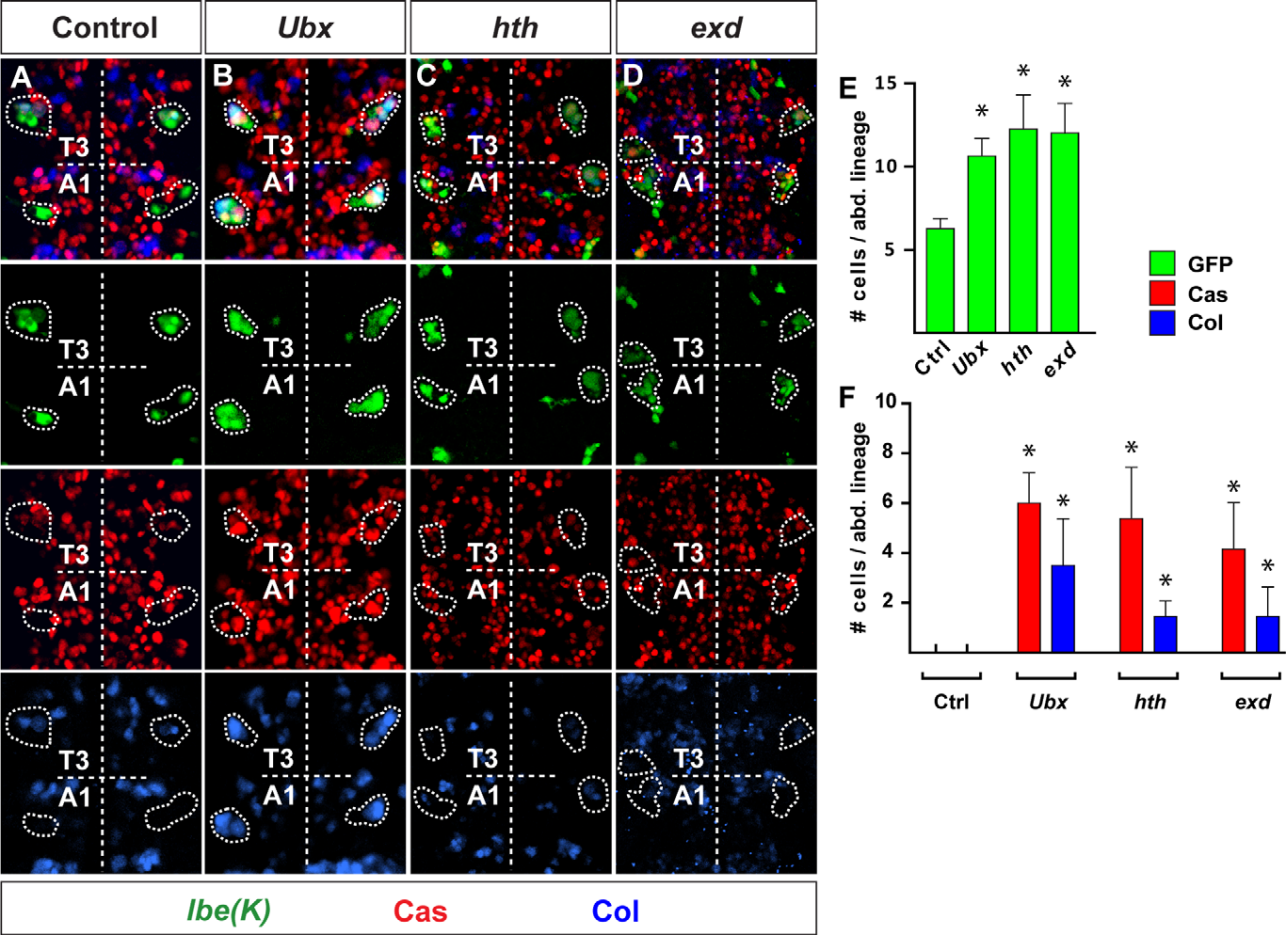
Suppression of thoracic-like NB 5–6 lineage progression by Ubx and Pbx/Meis. (A) At stage 15, *lbe(K)-Gal4* reveals a larger NB 5–6 lineage in thoracic segments, when compared to abdominal ones. Expression of Cas and Col is only evident in the NB 5-6T lineage. (B–D) At stage 15, in *Ubx*, *hth*, and *exd* mutants, NB 5-6A lineage is larger and expression of Cas and Col is evident. (E and F) Quantification of GFP, Cas, and Col positive cells/NB 5-6A lineage, in control, *Ubx*, *hth*, and *exd* mutants, at stage 14 (*n*>8 lineages). Asterisks denote significant difference compared to thoracic control (*p*<0.01, Student two-tailed test). Genotypes: (A) *lbe(K)-Gal4*, *UAS-nmEGFP/+*; *lbe(K)-Gal4*, *UAS-nmEGFP*/+. (B) *lbe(K)-Gal4,UAS-nmEGFP;Ubx*. (C) *lbe(K)-Gal4,UAS-nmEGFP;hth^5E04^/hth^Df3R^*. (D) *exd^B108^*, *FRT^18D^/y*; *lbe(K)-Gal4*, *UAS-nmEGFP*/+; *lbe(K)-Gal4*, *UAS-nmEGFP*/+.

The NB 5-6A lineage is smaller in size when compared to NB 5-6T. The Bx-C Hox genes are expressed at the proper time and place to be involved in this lineage truncation (Figure 2). Indeed, we find that mutations in Bx-C lead to the appearance of bona fide Ap clusters in more posterior regions, with the anticipated complexity due to their overlapping segmental expression levels and functions (Figure 5). Focusing on *Ubx* and the A1 segment, we utilized the *lbe(K)-Gal4* marker to address cell numbers in the NB 5-6A lineage, and found that the lineage contains a larger number of cells—equivalent in size to NB 5-6T (Figure 6A, 6B, and 6E). The temporal gene *cas* and the Ap cluster determinant *col* are both expressed at the end of the NB 5-6T lineage, but are not normally expressed in the smaller NB 5-6A lineage (Figure 6A; Figure 2). As anticipated from the larger NB 5-6A lineage observed in *Ubx* mutants, we also find ectopic expression of Cas and Col (Figure 6A, 6B, 6E, and 6F). Conversely, we find that when we misexpress *Ubx* early in NB 5-6T lineage, using the *lbe(K)-Gal4* driver—a driver that will ensure strong *Ubx* expression specifically in NB 5–6 already at stage 11 (Figure 1A)—*Ubx* is sufficient to suppress thoracic lineage progression, resulting in an abdominal-sized lineage, and loss of Cas and Col expression (Figure S4A–S4C). Similar results were obtained misexpressing *abd-A* (unpublished data). In contrast, late postmitotic misexpression of *Ubx* in Ap cluster neurons, driven from the *ap^Gal4^* driver, revealed no effect upon Ap cluster specification (Figure S4D–S4F). Thus, Bx-C genes are necessary and sufficient to terminate the NB 5–6 lineage within the Pdm temporal window, and serve this function rapidly after onset of their expression.

### *Antp*, *hth*, and *exd* Play Critical Roles during Thoracic NB 5–6 Development

In *Drosophila*, the thoracic segments, and in particular T2, have sometimes been viewed as a “ground state” of development, i.e., in the absence of all Hox gene input, abdominal and thoracic segments develop into a rudimentary T2 segment [46]. On this note, it was interesting to address how the NB 5-6T lineage would develop in an *Antp* mutant. To our surprise, we found a complete absence of Ap clusters in *Antp* mutants, as evident by the complete loss of the determinants Col, *ap^lacZ^*, Eya, Dac, and Dimm, as well as terminal identity markers: the neuropeptides Nplp1 and FMRFa (Figure S5). However, the *lbe(K)-lacZ* marker revealed that the lineage still progressed, and the two Ap neuron determinants *squeeze* (*sqz*) and Nab were not down-regulated (Figure S5I, S5J, S5Q, S5R, S5X, and S5Z).

Hox genes often act genetically and physically with the two Hox cofactors Hth and Exd [11],[41], and we therefore anticipated similar effects in these two mutants when compared to *Antp*. Indeed, we find that both *hth* and *exd* mutants fail to properly specify Ap neurons, as evident by the complete loss of Nplp1, FMRFa, *ap^lacZ^*, Eya, and Dimm, as well as the partial loss of Dac and Col expression (Figure S5). Similar to *Antp* mutants, the *lbe(K)-lacZ* marker revealed that the lineage still progressed, and *sqz* and Nab were not down-regulated in *hth* or *exd* mutants (Figure S5K, S5L, S5S, S5T, S5X, and S5Z).

**Figure 7:**
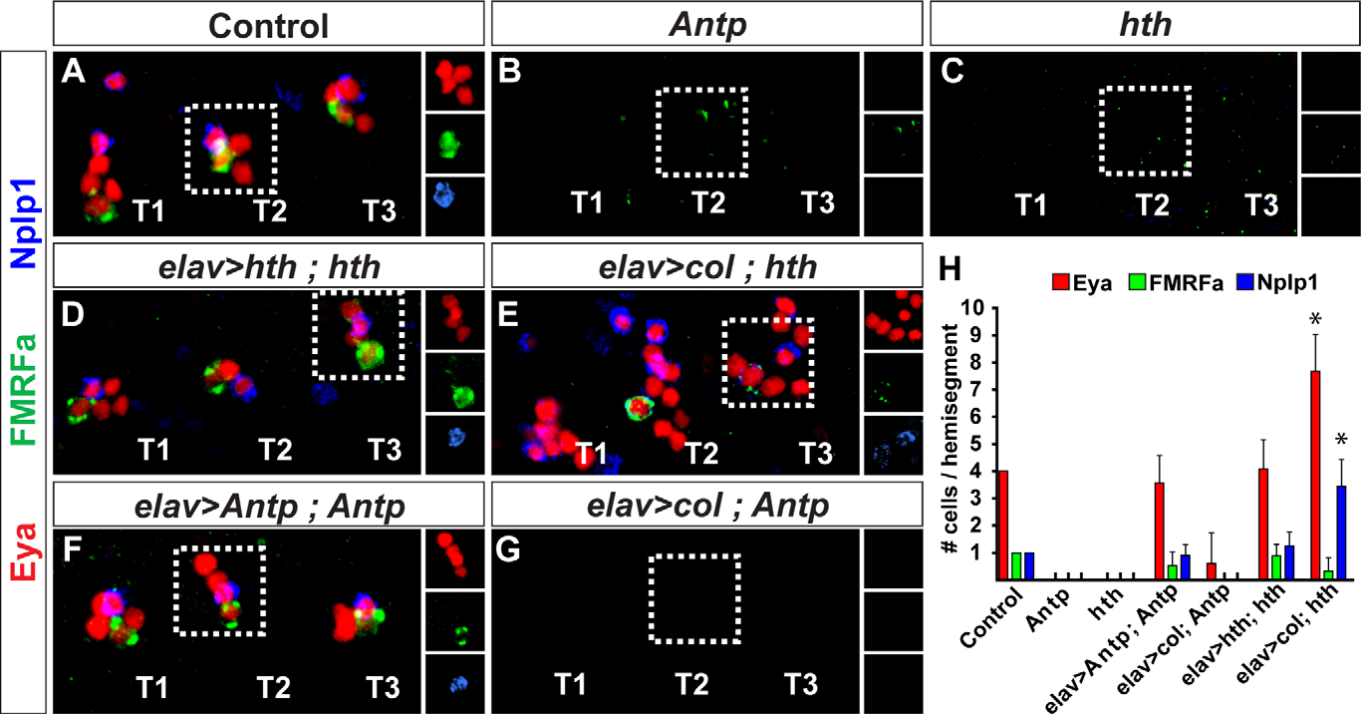
*collier* is able to rescue *homothorax*, but not *Antp*. (A–G) Expression of Eya, FMRFa, and Nplp1 in thoracic segments, at 18 h AEL. Small boxes show the expression of Eya, Nplp1, and proFMRFa in separate panels. (A–C) In control (A), Ap clusters are present, whereas in *Antp* (B) and *hth* (C) mutants, expression of Eya, FMRFa, and Nplp1 is lost. (D) Rescue of *hth* by *hth* and (F) of *Antp* by *Antp*. Both rescues restore Ap-clusters, as evident from the expression of Eya, FMRFa, and Nplp1. In contrast, whereas cross-rescue of *hth* by *col* is successful (E), cross-rescue of *Antp* by *col* does not restore Ap clusters (G). The extra Ap cluster neurons observed in cross-rescue of *hth* by *col* (E) is an effect of ectopic *col* in the early NB 5–6 lineage when using this *Gal4* driver [25]. (H) Quantification of Eya, Nplp1, and FMRFa positive cells/hemisegment (*n*>20 hemisegments). Asterisk denote significant difference compared to *hth* rescue, (*p*<0.01, Student two-tailed test). Genotypes: (A) *w^1118^*. (B) *Antp^25^/Antp^Ns-rvC12^*. (C) *hth^5E04^/hth^Df3R^*. (D) *UAS-hth/+*; *elav-Gal4,hth^5E04^/hth^Df3R^*. (E) *UAS-col/+*; *elav-Gal4,hth^5E04^/hth^Df3R^*. (F) *UAS-Antp/+*; *elav-Gal4,Antp^14^/Antp^Ns-rvC12^*. (G) *UAS-col/+*; *elav-Gal4,Antp^14^/Antp^Ns-rvC12^*.

The loss of the key Ap neuron determinant Col in *Antp*, *hth*, and *exd* mutants prompted us to ask the question of whether or not the primary function of *Antp*, *hth*, and *exd* may be to activate *col*. If so, it should be possible to rescue *Antp*, *hth*, and *exd* with *col*. This experiment was not technically feasible for *exd*, due to its maternal contribution, but was conducted for *Antp* and *hth*. For *hth*, we indeed find a restricted role, and cross-rescue of *hth* with *col* restores Ap clusters (Figure 7A, 7C–7E, and 7H). In contrast, *Antp* is not rescued by *col* (Figure 7A, 7B, and 7F–7H).

We thus find that *Antp*, *hth*, and *exd* play some common roles during NB 5-6T development, such as the regulation of *col*. But whereas *hth* can be cross-rescued by *col*, *Antp* cannot. The failure of *col* to rescue *Antp* suggests that *Antp* plays additional roles during Ap neuron specification.

### *homothorax* Acts in a Temporal Manner to Activate *collier* within the Castor Window

The temporal gene *cas* plays a key role in regulating many Ap neuron determinants, including *col*. However, the complete loss of Ap cluster determinants in *cas* mutants can largely be cross-rescued by re-expression of *col* [35]. But Cas is expressed already at stage early 11, and generates 6 cells prior to activating the Ap window. Moreover, whereas the initiation of the Ap window coincides with Grh expression (Figure 2B), *grh* mutants still generate Ap clusters with normal Ap1/Nplp1 and Ap2/3 neurons [35]. This indicates the existence of an unknown critical cue, acting within the *cas* window to trigger the Ap window, i.e., activating *col*.

**Figure 8:**
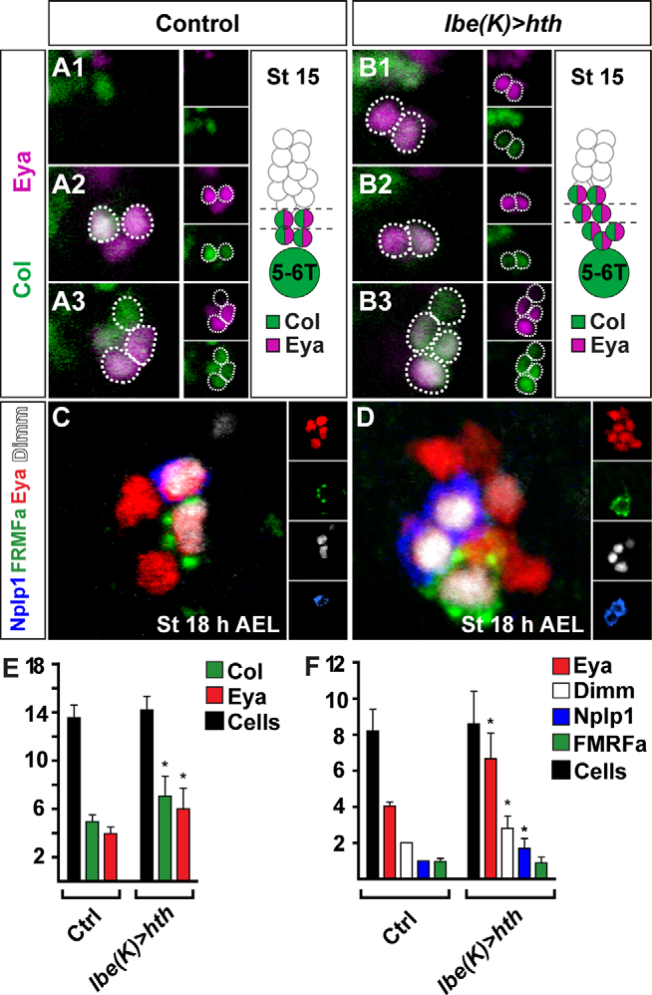
Overexpression of *homothorax* triggers the Ap window by activating *collier*. (A and B) Images showing expression of Col and Eya within the dorsal (A1 and B1), intermediate (A2 and B2), and ventral (A3 and B3) part of the NB 5-6T lineage; shown are top views, with anterior up. Cartoons represent the NB 5-6T lineage, based upon lineage mapping data [35]. Small boxes show the expression of Eya and Col in separate panels. In control, Ap cluster neurons coexpress Col and Eya at stage 15 and occupy intermediate (A2) and ventral (A3) layers, close to the neuroblast. In *hth* overexpression embryos, ectopic Col and Eya expressing cells appear in the intermediate (B2) and dorsal (B1) layers of the NB 5-6T lineage. (C and D) Expression of Eya, Dimm, Nplp1, and FMRFa within single T2 Ap clusters at stage 18 h AEL; shown are side views, with anterior to the left. Small boxes show the expression of Eya, Dimm, Nplp1, and proFMRFa in separate panels. (C) In control, Eya is specifically expressed within the four Ap cluster neurons, and Nplp1/Dimm and FMRFa/Dimm are expressed within Ap1/Nplp1 and Ap4/FMRFa neurons, respectively. (D) When *hth* is overexpressed within the NB 5-6T lineage, extra Eya, Dimm, and Nplp1 expressing cells appear. (E) Quantification (*n*>8 VNCs) of Col and Eya cell numbers, at stage 15, and NB 5-6 T lineage cell numbers, at stage 14. (F) Quantification (*n*>8 VNCs) of Eya, Nplp1, Dimm, FMRFa, and NB 5-6 T lineage cell numbers at stage 18 h AEL. Values as mean number of expressing cells within single NB 5-6T lineages, error bars show SD. Asterisks (*) denote significant difference compared to control (*p*<0.01, Student two-tailed *t*-test). Genotypes: (A, C, and E) *lbe(K)-Gal4*/+. (B, D, and F) *UAS-hth*/+; *lbe(K)-Gal4*/+.

In *hth* mutants, there is a failure of Ap neuron specification, evident from the loss or reduction of Col, *ap^lacZ^*, Eya, Dimm, Nplp1, and FMRFa expression (Figure S5). However, similar to *cas*, Ap clusters can be rescued simply by re-expressing *col*, indicating that the primary role of *hth* is to activate *col*. Is *hth* then the critical trigger, acting in the large Cas window to trigger the Ap window by activating *col*? Our expression analysis argued against this idea, since we found that Hth is indeed present in the NB 5-6T lineage already at stage 11 (Figure 2B), two stages prior to onset of Col expression. However, the answer seems to lie in the actual levels of Hth—as mentioned earlier, we noticed that its expression was weak at stage 11, with a sharp increase at stages 12–13, preceding the onset of *col* expression (Figure 3). To test whether increasing levels of Hth is sufficient to trigger the Ap window, we overexpressed *hth* using the *lbe(K)-Gal4* driver—a driver that will ensure strong *hth* expression specifically in NB 5–6, already at stage 11 (Figure 1A). Strikingly, *hth* overexpression triggered premature Col expression in the NB 5-6T lineage (Figure 8A, 8B, and 8E). As anticipated from this effect, we also noticed a robust increase in the number of Ap neurons specified within the NB 5-6T lineage, as evident by ectopic expression of Eya, Nplp1 and FMRFa (Figure 8C, 8D, and 8F). Quantification of NB 5-6T cell numbers, at stage 14 and 18 h after egg laying (AEL), revealed no increase in cell numbers, either at stage 14—showing that neither GMCs nor Ap neurons are dividing erroneously—or at late stages—showing that the neuroblast is not continuing to divide past stage 15 (Figure 8E and 8F). Thus, *hth* overexpression results in Ap neuron specification of cells born in the early Cas window, but does not trigger extra cell divisions in any part of the lineage.

Within the large Cas window, a switch from low- to high-level Hth appears to function as a critical temporal switch, which together with Cas, Antp, and Exd, acts to trigger Col expression. Col in turn specifies Ap neurons by activating a critical feed-forward loop [25]. Thus, within this particular CNS lineage, one critical integration point between anteroposterior and temporal cues is the activation of the COE/Ebf regulator *col* and its feed-forward loop.

### Early versus Late Rescue of *homothorax* Reveals its Different Roles

**Figure 9:**
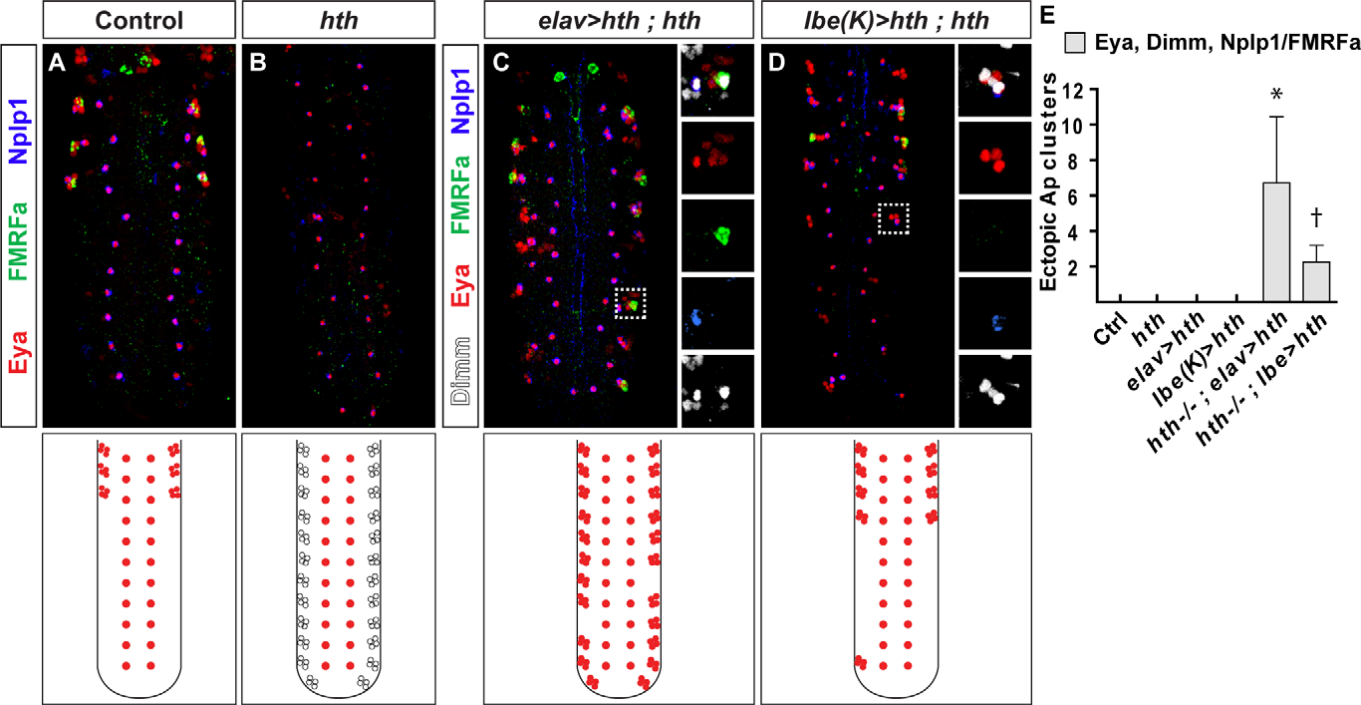
Rescue of *homothorax* at different time points reveals its dual role. (A and B) Control and *hth* VNCs reveal the loss of differentiated Ap cluster neurons in *hth* mutants. However, as shown above, in *hth* mutants, Ap cluster cells are still present in NB 5-6T. In the abdomen, *hth* mutants fail to truncate the NB 5-6A lineage, resulting in the appearance of Ap cluster cells. (C and D) Rescue of *hth* using a late (C) and an early (D) *Gal4* driver reveals the dual role of *hth*. Small boxes show the expression of Eya, Nplp1, and proFMRFa in separate panels. (C) Late expression allows for *hth* to play its late role in Ap cluster neuron specification, and Ap clusters appear in the majority of abdominal segments, evident by the expression of Eya, Nplp1, and FMRFa. However, using this *Gal4* driver, *hth* is reintroduced too late to truncate the NB 5-6A lineage. (D) In contrast, early expression of *hth* reintroduces *hth* early enough to truncate the NB 5-6A lineage, evident by fewer Ap clusters in the abdomen. In addition, in the thoracic segments, *hth* still can play its late role—specifying Ap cluster neurons—evident by the reappearance of Ap clusters in thoracic segments. (E) Quantification of Ap clusters, defined as the presence of Eya, Dimm, and at least one of the two neuropeptides FMRFa and Nplp1 in abdominal lateral segments (abdominal Ap clusters/VNC; *n*>7). Asterisk denotes significant difference compared to control, † denotes significant difference compared to *elav>hth*; *hth^−/−^* (*p*<0.01; Student two-tailed test). Genotypes: (A) *w^1118^*. (B) *hth^5E04^/hth^Df3R^*. (C) *UAS-hth/+*; *elav-Gal4,hth^5E04^/hth^Df3R^*. (D) *lbe(K)-Gal4/UAS-hth;hth^5E04^/hth^Df3R^*.

Are the roles of the Bx-C genes in lineage termination in the NB 5-6A lineage also dependent upon the Pbx/Meis factors? That appears to be the case: similar to *Ubx*, both *hth* and *exd* mutants display an increase in NB 5-6A lineage cell numbers, approaching those normally found in NB 5-6T, as well as ectopic expression of Cas and Col (Figure S6C–S6F). But why do the Bx-C and Pbx/Meis mutants display such different phenotypes—when assayed using late Ap neuron markers, Bx-C mutants display striking homeotic transformations, with bona fide Ap clusters generated throughout the VNC (Figure 5). In contrast, *hth* and *exd* mutants display a complete loss of Ap cluster specification (Figure S5). The answer to this paradox comes from the dual role of *hth* and *exd* outlined above—these genes not only control lineage termination of the NB 5-6A lineage, but also specify Ap neurons in the larger NB 5-6T lineage. Thus, we reasoned that in *hth* and *exd* mutants, “Ap cluster cells” are likely present in abdominal segments, but are not properly specified into Ap cluster neurons due to the second and later role of *hth* and *exd*. To reveal this dual role, we focused on *hth* and attempted to rescue *hth* with itself, but at different stages of NB 5–6 lineage progression. Specifically, because NB 5-6A exits the cell cycle at stage 12, we sought to reintroduce *hth* expression before versus after this exit point. To this end, we used the stage 11 driver *lbe(K)-Gal4* versus the stage 12 driver *elav-Gal4* (Figure 1A; Figure S6). The prediction was that if *hth* was rescued by itself at a later stage, then posterior, ectopic Ap cluster cells would be triggered to differentiate into bona fide Ap cluster neurons, and this rescue would therefore phenocopy Bx-C mutants. This is indeed what we find: late rescue of *hth* not only restores Ap clusters in thoracic segments, but results in ectopic Ap clusters also in the majority of abdominal segments (Figure 9A–9C and 9E). In contrast, if *hth* was rescued earlier in the lineage, prior to the neuroblast cell cycle exit point, *hth* would be able to play both its early role—blocking NB 5-6A cell cycle in the abdomen—and its late role—specifying Ap neurons in the thorax. Thus, we predicted that early rescue would reveal a more complete rescue of *hth*, with Ap clusters only in the thoracic segments. As anticipated, this is what we find, as evident from robust rescue of Ap clusters in thoracic segments, but with reduced prevalence of ectopic abdominal clusters (Figure 9D and 9E).

Thus, low-level Hth is essential in NB 5-6A prior to stage 12 to ensure cell cycle exit. If this critical stop-point is bypassed, subsequent reintroduction of Hth is not able to halt the lineage progression at any later point, but will, however, allow Hth to act in its late cell specification role, i.e., activating *col* and thereby specifying generic Ap neurons. This dual role of *hth*—acting early with Bx-C genes in abdominal segments to restrict lineage size, and with *Antp* in thoracic segments to specify Ap cluster neurons—is revealed by *Gal4/UAS*-mediated rescue at different stages of NB 5–6 lineage progression.

### *Antp* Can Trigger Partial Ap Clusters in Anterior NB 5–6 Lineages

**Figure 10:**
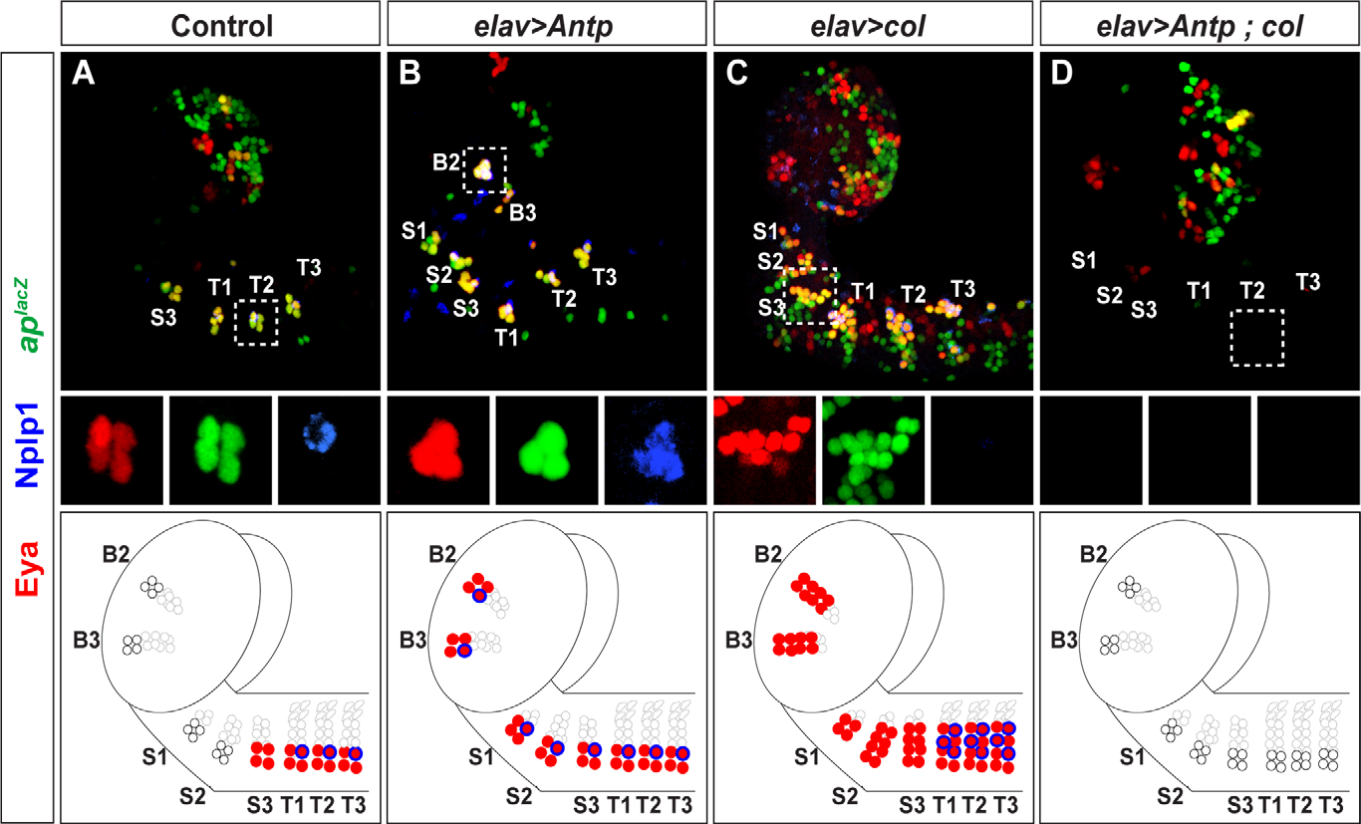
Misexpression of *Antp* triggers Ap cluster formation in anterior NB 5–6 lineages. (A and B) Compared to control (A), pan-neuronal misexpression of *Antp* (B) triggers ectopic formation of Ap-clusters into brain segment B2, as evident by the coexpression of Eya, *ap^lacZ^*, and Nplp1. Small boxes show the expression of Eya, Nplp1, and *ap^lacZ^* expression in separate panels. (C) Misexpression of *col* triggers ectopic Eya and *ap^lacZ^* expression throughout the anterior CNS. However, despite extensive Eya/*ap^lacZ^* coexpression, we find no evidence of Nplp1 expression, confirming the notion that *Antp* plays additional roles beyond activating *col*. (D) Misexpressing *Antp* in a *col* mutant background, reveals a complete loss of Ap clusters in thoracic segments (the *col* mutant phenotype), and a failure to trigger ectopic anterior Ap cluster, evident by the absence of Eya/*ap^lacZ^*/Nplp1 expressing clusters, demonstrating that *Antp* requires *col* to trigger Ap cluster formation. Genotypes: (A) *ap^lacZ^;elav-Gal4/+*. (B) *ap^lacZ^/UAS-Antp;elav-Gal4/+*. (C) *ap^lacZ^/UAS-col;elav-Gal4/+*. (D) *col^3^*, *ap^lacZ^/col^1^*; *elav-Gal4/UAS-Antp*.

**Figure 11:**
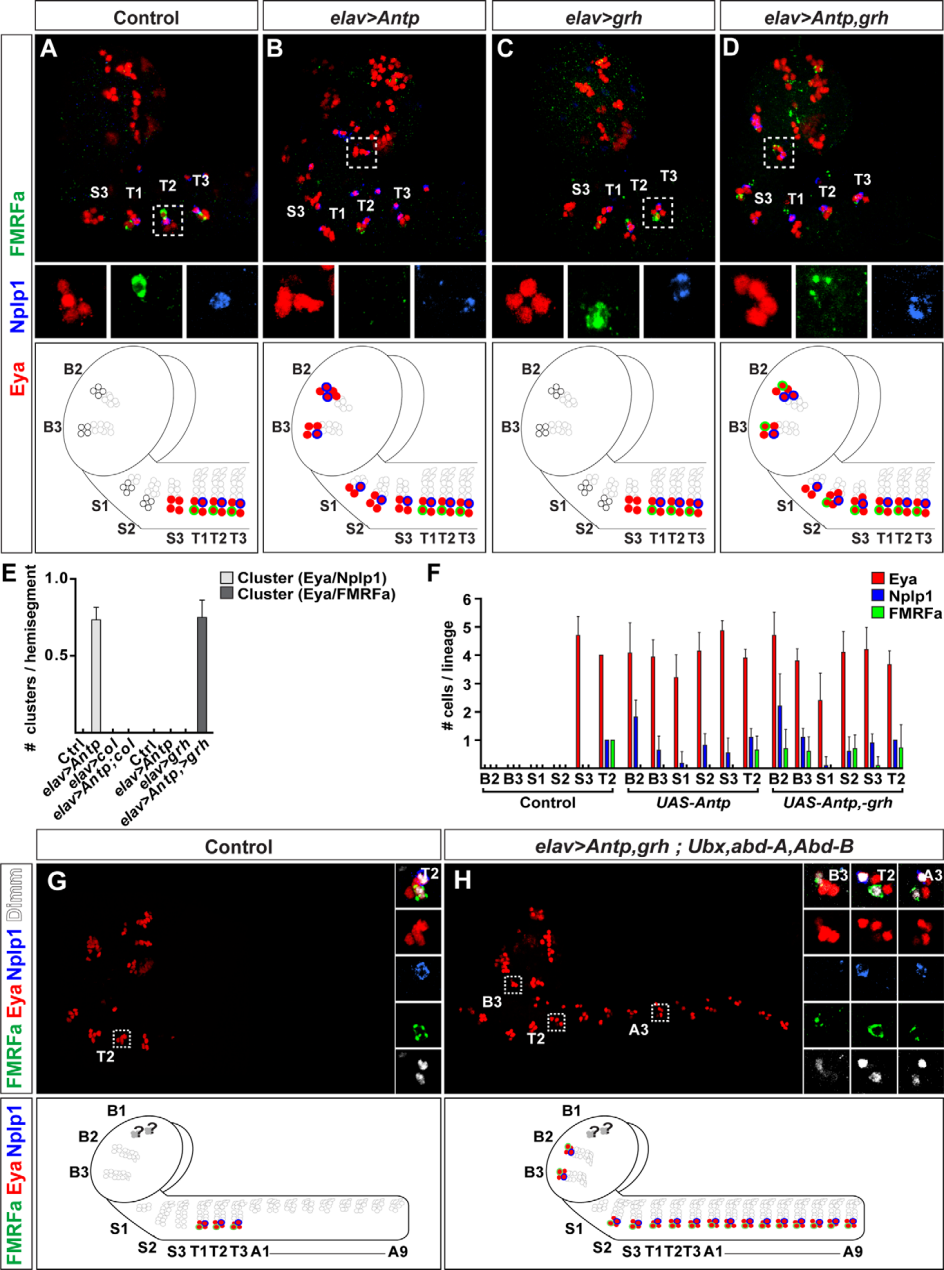
Comisexpression of *Antp* with *grainyhead* triggers complete Ap cluster specification in anterior NB 5–6 lineages. (A and B) Compared to control (A), misexpression of *Antp* (B) triggers partial Ap clusters, evident by anterior Eya/Nplp1 clusters. However, there is no ectopic expression of FMRFa. Small boxes show the expression of Eya, Nplp1, and proFMRFa expression in separate panels. (C) Misexpression of *grh* does not trigger ectopic Ap clusters, evident by absence of Eya/Nplp1 cluster. (D) However, co-misexpression of *Antp* and *grh*, leads to the appearance of ectopic anterior Ap clusters, evident by the coexpression of Eya/Nplp1 with FMRFa. (E) Quantification of the number of Ap clusters/hemisegment, combined for B2–S3, as defined by the presence of Eya/Nplp1 and Eya/FMRFa (clusters/hemisegment; *n*>10 CNSs). (F) Individual quantification of the number of cells expressing Eya, Nplp1, and FMRFa, in each of the six brain segments (B2–S3), and the thoracic cluster T2 (cells/lineage, *n*>10). (G) Control and (H) *Antp*, *grh* co-misexpression in *Ubx*, *abd-A*, *Abd-B* triple mutant. In wild type (G), Ap clusters are confined to the three thoracic segments. Small boxes show the expression of Eya, Dimm, Nplp1, and proFMRFa in separate panels. (H) In triple Bx-C mutants that co-misexpress *Antp* and *grh*, Ap clusters form throughout the neuroaxis. (G and H) are composed from multiple images. Genotypes: (A) *ap^lacZ^;elav-Gal4/+*. (B) *ap^lacZ^/UAS-Antp;elav-Gal4/+*. (C) *ap^lacZ^/UAS-grh;elav-Gal4/+*. (D) *ap^lacZ^/UAS-Antp,UAS-grh;elav-Gal4/+*. (E and F) Genotypes as in (A–D). (G) *C155-Gal4*/+. (H) *C155-Gal4/y*; *UAS-Antp*, *UAS-grh/+*; *Ubx*, *abd-A*, *Abd-B*.

How is the NB 5–6 lineage modified in more anterior segments? Analyzing anterior NB 5–6 lineages, we found a significant degree of variation with respect to lineage size. However, expression of the temporal factor Cas was observed in all segments (Figure S7). Cas is expressed at the end of the thoracic NB 5–6 lineage (Figure 2B), and plays a critical role to activate Ap neuron determinants [35]. Cas is not expressed in the abdominal lineage, since the abdominal NB 5–6 lineage terminates just prior of progression into the Cas window (Figure 2B). However, the presence of NB 5–6 lineages anteriorly, containing a Cas window, suggested that Ap cluster neuron equivalents may indeed be present in anterior segments. *Antp* plays a critical role for Ap neuron specification, but its expression stops at the T1 segment (Figure 2A). Therefore, we postulated that anterior misexpression of Antp may be sufficient to specify ectopic anterior Ap clusters. We confirmed this notion, as evident by the appearance of *ap^lacZ^*, Eya, and Nplp1 expression in anterior segments (Figures 10A, 10B, and 11E). To verify that these ectopic Ap clusters indeed were generated from anterior NB 5–6 equivalents, we utilized the NB 5–6–specific driver *lbe(K)-Gal4* to misexpress *Antp*, and could again identify ectopic anterior Ap clusters (Figure S8). In the anterior-most segments, B1 and B2, there is added complexity due to more extensive expression of both *ap^lacZ^* and Eya already in the wild type, and the presence of *ap^lacZ^*/Eya coexpressing cells (Figures 10A and 11E). However, these cells do not coexpress Nplp1, nor do they stem from anterior NB 5–6 lineages (Figures 10A; unpublished data). Thus, we were able to identify ectopic Ap clusters in the B2 segment (Figure 10B). However, we found no ectopic Ap clusters in the B1 segment (Figure 10B).

Our analysis of the function of *Antp* and *col* in the NB 5-6T, demonstrated that while *Antp* plays a key role in activating *col*, Antp also plays additional roles to specify Ap cluster neurons (Figure 7). In line with these findings, we find that whereas misexpression of *col* is able to act in the non-Antp domain to trigger ectopic anterior expression of both *ap^lacZ^* and Eya, it is not able to trigger formation of bona fide anterior Ap clusters, as evident by the failure to activate Nplp1 and FMRFa (Figures 10C and 11E; unpublished data). Similarly, *Antp* is unable to trigger ectopic, anterior Ap clusters in a *col* mutant background (Figure 10D). Thus, the regulatory interplay observed between *Antp* and *col* in the NB 5-6T lineage is recapitulated anteriorly, in the ectopic setting.

We found no evidence of regulatory interplay between *Antp*, *hth*, *exd*, and the late temporal genes *cas* and *grh* (Figure S9). In line with this notion, we do not find any evidence of a complete homeotic transformation by *Antp* of anterior NB 5–6 lineages, since the total number of cells in the lineage, as well as the number of Cas and Grh cells, are unaffected by *Antp* misexpression (Figure S10). Thus, our results support the notion that these effects of *Antp* misexpression occur postmitotically and are not due to complete homeotic transformation of anterior NB 5–6 lineages.

### Antp and Grh Cooperate to Trigger Bona Fide Ap Clusters in Anterior NB 5–6 Lineages

Misexpression of *Antp* triggers ectopic Ap clusters in anterior NB 5–6 lineages, with the expression of the Nplp1 neuropeptide. However, we failed to detect FMRFa neuropeptide in these ectopic Ap clusters (Figure 11A, 11B, and 11E). Ap neurons are generated at the end of the NB 5-6T lineage, in a temporal window that in addition to *cas*, also expresses the *grh* temporal gene, i.e., in a Cas/Grh coexpressing window (Figure 2B). Whereas *cas* plays a global role at the end of the NB 5-6T lineage, regulating most Ap neuron determinants, *grh* plays a more selective role, and at high levels, is necessary and sufficient to specify the last-born cell, the FMRFa neuron [35]. The failure of *Antp* to trigger FMRFa expression in the anterior ectopic Ap clusters prompted us to examine the expression of Grh in anterior NB 5–6 lineages. This analysis revealed that there is indeed weak or no expression of Grh in anterior NB 5–6 lineages (Figure S7). We next tested whether or not ectopic expression of *grh* alone could trigger ectopic anterior Ap cluster neurons, with Nplp1 and FMRFa expression. However, given the lack of Antp expression in anterior segments, we were not surprised to find that *grh* misexpression did not to trigger ectopic Ap clusters (Figure 11C and 11E). Therefore, we postulated that by co-misexpressing *Antp* with *grh*, we should be able to trigger the appearance of ectopic Ap clusters with a more complete identity, i.e., with expression not only of Nplp1, but also of FMRFa. This is indeed what we find (Figure 11D and 11E). To verify that these ectopic Ap clusters indeed were generated from anterior NB 5–6 equivalents, we utilized the NB 5–6–specific driver *lbe(K)-Gal4* to misexpress *Antp* and *grh*, and could again identify ectopic anterior Ap clusters (Figure S8). However, we were again unable to trigger Ap clusters in the B1 segment (unpublished data).

Whereas many of the posterior *Drosophila* CNS segments, such as A2–A7, are generally viewed as identical, repetitive units, all brain segments (B1–B3 through S1–S3) are considered unique [47]. Our lineage analysis of anterior wild-type NB 5–6 lineages confirmed this notion, revealing that both lineage size, as well as the expression of Cas and Grh, is different between segments (Figure S10). Intriguingly, we also find that the effects of *Antp* misexpression, as well as *Antp*/*grh* co-misexpression, resulted in different types of ectopic Ap clusters in different brain segments, with reproducibly distinct numbers of Eya, Nplp1, and FMRFa neurons (Figure 7F). These findings suggest that *Antp*/*grh* co-misexpression is not able to override all aspects of segment specificity within anterior NB 5–6 lineages.

### Generation of a “Thoracic CNS”

In Bx-C mutants, we find homeotic transformation of abdominal segments into a thoracic identity, with ectopic Ap clusters in each segment. When we co-misexpress *Antp* and *grh*, we find ectopic Ap clusters in anterior segments. We reasoned that by performing both of these genetic manipulations simultaneously, we would be able to trigger formation of a “thoracic CNS,” i.e., a CNS containing Ap clusters along the entire neuroaxis. This is indeed what we found: co-misexpression of *Antp* and *grh*, in a *Ubx*, *abd-A*, *Abd-B* triple mutant background, resulted in ectopic Ap clusters along the neuroaxis, evident by expression of Eya, Nplp1, and FMRFa in all segments (Figure 11G and 11H). Again, the anterior-most segment, B1, did not display ectopic Ap clusters.

## Discussion

**Figure 12:**
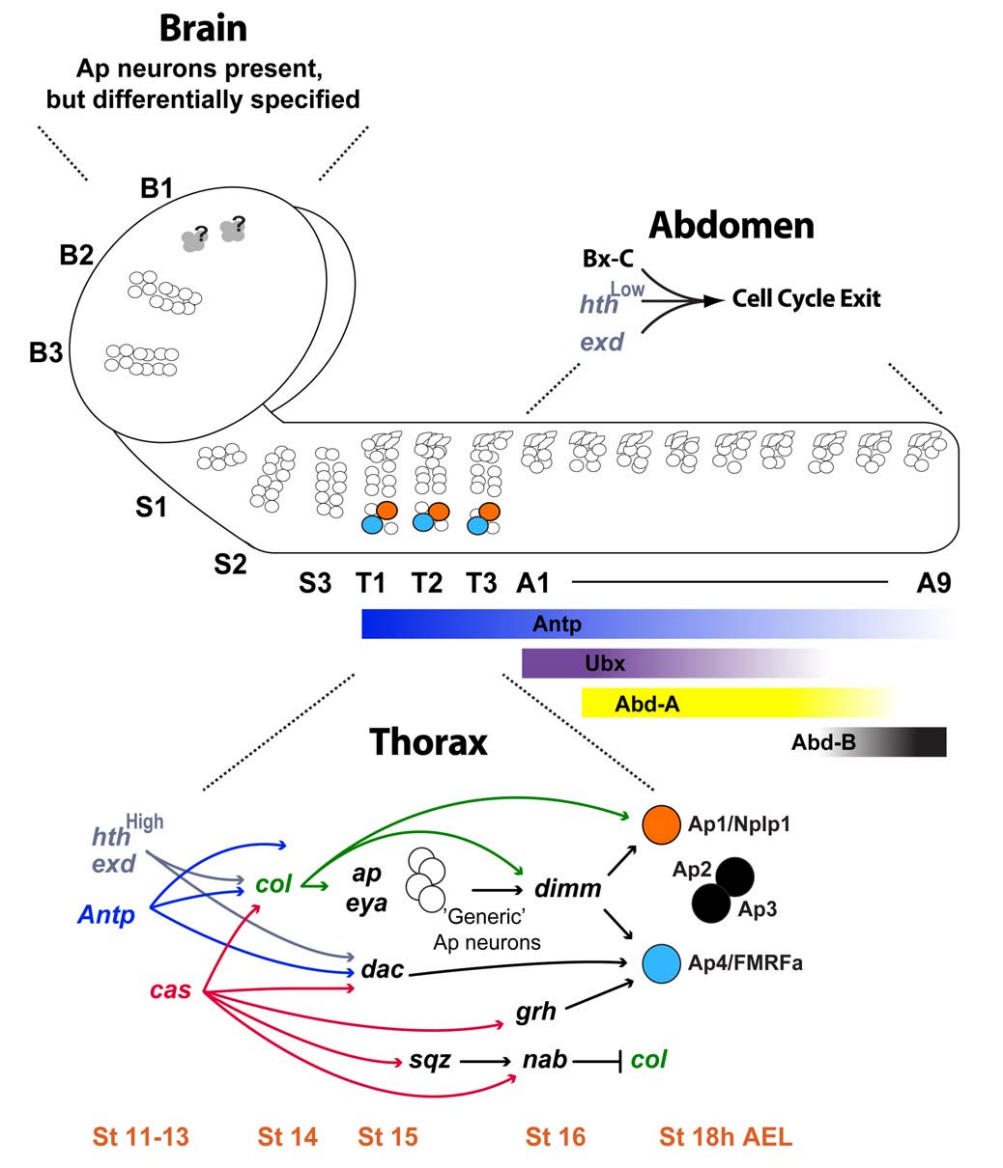
Summary of Hox/Pbx/Meis and temporal control of NB 5–6 development. The NB 5–6 lineage is generated in all CNS segments, but the genetic pathway leading to Ap cluster formation is only triggered in the NB 5-6T lineages. Three separate mechanisms act to ensure this segment-specific event. In abdominal segments, the Pbx/Meis genes *hth* and *exd* act with Bx-C Hox genes to truncate the NB 5-6A lineage by triggering neuroblast cell cycle exit within an early temporal (Pdm) window. This occurs prior to generation of Ap cluster cells, and prior to progression into the Cas/Grh late temporal window. In thoracic segments, the absence of Bx-C expression in the NB 5-6T neuroblast allows it to progress further and generate a larger lineage, thereby generating the Ap cluster cells. Importantly, this also allows for the lineage to progress into the Cas/Grh late temporal window. Combined with the expression of the thoracic Hox gene *Antp*, and increasing levels of Hth, this allows for integration of anteroposterior and temporal cues and the specification of Ap cluster cells into Ap cluster neurons, primarily by the activation of the critical Ap cluster determinant *col*. Grh plays a postmitotic role in specifying the Ap4/FMRFa cell fate. In anterior segments, the NB 5–6 lineage, although varying in size when compared to thoracic segments, does contain a Cas window. However, the absence of *Antp* expression, coupled with weak or absent expression of the late temporal gene *grh*, prevents specification of Ap cluster neurons.

To understand segment-specific neuronal subtype specification, we have focused on the *Drosophila* neuroblast 5–6 lineage and the thoracic-specific Ap cluster neurons born at the end of the NB 5-6T lineage. We find that the thoracic appearance of Ap clusters results from a complex interplay of Hox, Pbx/Meis, and temporal genes that act to modify the NB 5–6 lineage in three distinct ways (Figure 12). In line with other studies of anterior-most brain development, we find that the B1 segment appears to develop by a different logic. We will discuss these findings in relation to other studies on spatial and temporal control of neuroblast lineages.

### Abdominal Segments: Lineage Truncation

In the developing *Drosophila* CNS, each abdominal and thoracic hemisegment contains an identifiable set of 30 neuroblasts, which divide asymmetrically in a stem-cell fashion to generate distinct lineages. However, they generate differently sized lineages—from two to 40 cells [19],[20]—indicating the existence of elaborate and precise mechanisms for controlling lineage progression. Moreover, about one third of these lineages show reproducible anteroposterior differences in size, typically being smaller in abdominal segments when compared to thoracic segments [19]–[21],[48]. Thus, neuroblast-specific lineage size control mechanisms are often modified along the anteroposterior axis.

Previous studies have shown that Hox input plays a key role in modulating segment-specific behaviors of neuroblast lineages [49]. Recent studies have resulted in mechanistic insight into these events. For instance, in the embryonic CNS, Bx-C acts to modify the NB 6-4 lineage, preventing formation of thoracic-specific neurons in the abdominal segments. This is controlled, at least in part, by Bx-C genes suppressing the expression of the *Cyclin E* cell cycle gene in NB 6-4a [50]–[52]. Detailed studies of another neuroblast, NB 7-3, revealed that cell death played an important role in controlling lineage size in this lineage: when cell death is genetically blocked, lineage size increased from four up to 10 cells [53],[54]. Similarly, in postembryonic neuroblasts, both of these mechanisms have been identified. In one class of neuroblasts, denoted *type I*, an important final step involves nuclear accumulation of the Prospero regulator [55], a key regulator both of cell cycle and differentiation genes [56]. In “type II” neuroblasts, *grh* acts with the Bx-C gene *Abd-A* to activate cell death genes of the RHG family, and thereby terminates lineage progression by apoptosis of the neuroblast. This set of studies demonstrates that lineage progression, in both embryonic and postembryonic neuroblasts, can be terminated either by neuroblast cell cycle exit or by neuroblast apoptosis. In the abdominal segments, we find that the absence of Ap clusters results from a truncation of the NB 5–6 lineage, terminating it within the Pdm early temporal window, and therefore Ap cluster cells are never generated. Our studies reveal that this truncation results from neuroblast cell cycle exit, controlled by Bx-C, *hth*, and *exd*, thereafter followed by apoptosis. In Bx-C/*hth*/*exd* mutants, the neuroblast cell cycle exit point is bypassed, and a thoracic sized lineage is generated, indicating that these genes may control both cell cycle exit and apoptosis. However, it is also possible that cell cycle exit is necessary for apoptosis to commence, and that Bx-C/*hth*/*exd* in fact only control cell cycle exit. Insight into the precise mechanisms of the cell cycle exit and apoptosis in NB 5-6A may help shed light on this issue.

Whichever mechanism is used to terminate any given neuroblast lineage—cell cycle exit or cell death—the existence in the *Drosophila* CNS of stereotyped lineages progressing through defined temporal competence windows allows for the generation of segment-specific cell types simply by regulation of cell cycle and/or cell death genes by developmental patterning genes. Specifically, neuronal subtypes born at the end of a specific neuroblast lineage can be generated in a segment-specific fashion “simply” by segmentally controlling lineage size. This mechanism is different in its logic when compared to a more traditional view, where developmental patterning genes act upon cell fate determinants. But as increasing evidence points to stereotypic temporal changes also in vertebrate neural progenitor cells [12], this mechanism may well turn out to be frequently used to generate segment-specific cell types also in the vertebrate CNS.

### Thoracic Segments: Specification of Ap Cluster Neurons by Integration of Hox, Pbx/Meis, and Temporal Gene Function

Our findings of Hox, Pbx/Meis, and temporal gene input during Ap cluster formation is not surprising—generation and specification of most neurons and glia will, of course, depend upon some aspect or another of these fundamental cues. Importantly however, the detailed analysis of the NB 5-6T lineage, and of the complex genetic pathways acting to specify Ap cluster neurons, has allowed us to pin-point critical integration points between anteroposterior and temporal input. Specifically, *cas*, *Antp*, *hth*, and *exd* mutants show striking effects upon Ap cluster specification, with effects upon expression of many determinants, including the critical determinant *col*. Whereas *Antp* plays additional feed-forward roles, and *exd* was not tested due to its maternal load, we found that both *cas* and *hth* mutants can be rescued by simply re-expressing *col* ([35]; this study). This demonstrates that among a number of possible regulatory roles for *cas*, *hth*, *Antp*, and *exd*, one critical integration point for these anteroposterior and temporal cues is the activation of the COE/Ebf gene *col*, and the *col*-mediated feed-forward loop. Both *col* and *ap* play important roles during *Drosophila* muscle development, acting to control development of different muscle subsets [57],[58]. Their restricted expression in developing muscles has been shown to be under control of both Antp and Bx-C genes [59],[60]. Molecular analysis has revealed that this regulation is direct, as Hox proteins bind to key regulatory elements within the *col* and *ap* muscle enhancers [59],[60]. The regulatory elements controlling the CNS expression of *col* and *ap* are distinct from the muscle enhancers [23],[59]–[61] (unpublished data), and it will be interesting to learn whether Hox, as well as Pbx/Meis and temporal regulatory input, acts directly also upon the *col* and *ap* CNS enhancers.

### Levels of Homothorax Play Instructive Roles

One particularly surprising finding pertains to the instructive role of Hth levels in NB 5-6T. At low levels, Hth acts in NB 5-6A to block lineage progression, whereas at higher levels, it acts in NB 5-6T to trigger expression of *col* within the large *cas* window. It is interesting to note that the *hth* mRNA and Hth protein expression levels increase rapidly in the entire anterior CNS (T3 and onward) (this study) [42],[43]. In addition, studies reveal that thoracic and anterior neuroblast lineages in general tend to generate larger lineages [19],[20],[49] and thus remain mitotically active for a longer period than abdominal lineages. On this note, it is tempting to speculate that high levels of Hth may play instructive roles in many anterior neuroblast lineages. In zebrafish, Meis3 acts to modulate Hox gene function, and intriguingly, different Hox genes require different levels of Meis3 expression [62]. In the *Drosophila* peripheral nervous system, expression levels of the Cut homeodomain protein play instructive roles, acting at different levels to dictate different dendritic branching patterns in different sensory neuron subclasses [63]. Although the underlying mechanisms behind the levels-specific roles of Cut [63], Meis3 [62] or Hth (this study) are unknown, it is tempting to speculate that they may involve alterations in transcription factor binding sites, leading to levels-sensitive binding and gene activation of different target genes.

The vertebrate members of the Meis family (Meis1/2/3, Prep1/2) are expressed within the CNS, and play key roles in modulating Hox gene function. Intriguingly, studies in both zebrafish [64],[65] and *Xenopus* [66]–[68] reveal that subsequent to their early broad expression, several members are expressed more strongly or exclusively in anterior parts of the CNS, in particular, in the anterior spinal cord and hindbrain. Here, functional studies reveal complex roles of the Meis family with respect to Hox gene function and CNS development. However, in several cases, studies reveal that they are indeed important for specification, or perhaps generation, of cell types found in the anterior spinal cord and/or hindbrain, i.e., anteroposterior intermediate neural cell fates [62],[66]–[70]. As we learn more about vertebrate neural lineages, it will be interesting to learn which Meis functions may pertain to postmitotic neuronal subtype specification, and which may pertain to progenitor cell cycle control.

### Anterior Segments: Specification into Different Cell Types

In anterior segments—subesophageal (S1–S3) and brain (B1–B3)—a more complex picture emerges where both the overall lineage size and temporal coding is altered, when compared to the thoracic segments. Specially, whereas all anterior NB 5–6 lineages do contain Cas expressing cells, expression of Grh is weak or absent from many Cas cells. The importance of this weaker Grh expression is apparent from the effects of co-misexpressing *grh* with *Antp*—misexpression of *Antp* alone is unable to trigger FMRFa expression, whereas co-misexpression with *grh* potently does so. It is unclear why anterior 5–6 lineages would express lower levels of Grh, since Grh expression is robust in some other anterior lineages (unpublished data).

In the B1 segment, we and others identify not one, but two NB 5–6 equivalents [14]. However, the finding of two NB 5–6 equivalents is perhaps not surprising, since the B1 segment contains more than twice as many neuroblasts as posterior segments [36]–[38]. Due to weaker *lbe(K)-lacZ* and -*Gal4* reporter gene expression, and cell migration, we were unable to map out these lineages. However, irrespective of the features of the B1 NB 5–6 lineages, we were unable to trigger bona fide Ap cluster formation by *Antp/grh* co-misexpression in B1. Together, these findings suggest that the B1 segment develops using a different modus operandi, a notion that is similar to development of the anterior-most part of the vertebrate neuroaxis, where patterning and segmentation is still debated [71],[72]. On that note, it is noteworthy that although Hox genes play key roles in specifying unique neuronal cell fates in more posterior parts of the vertebrate CNS [8],[9],[73],[74], and can indeed alter cell fates when misexpressed, the sufficiency of Hox genes to alter neuronal cell fates in the anterior-most CNS has not been reported—for instance, Hox misexpression has not been reported to trigger motoneuron specification in the vertebrate forebrain. Thus, in line with our findings that *Antp* is not sufficient to trigger Ap cluster neuronal fate in the B1 anterior parts, the anterior-most part of both the insect and vertebrate neuroaxis appears to be “off limits” for Hox genes.

### The Integration of Temporal and Anteroposterior Cues with NB 5–6 Identity

The Hox, Pbx/Meis, and temporal genes are necessary, and in part sufficient, to dictate Ap cluster neuronal cell fate. However, they only do so within the limited context of NB 5–6 identity. Within each abdominal and thoracic hemisegment, each of the 30 neuroblasts acquires a unique identity, determined by the interplay of segment-polarity and columnar genes [75],[76]. In the periphery, recent studies demonstrate that anteroposterior cues, mediated by Hox and Pbx/Meis genes, are integrated with segment-polarity cues by means of physical interaction and binding to regulatory regions of specific target genes [77]. It is tempting to speculate that similar mechanisms may act inside the CNS as well, and may not only involve anteroposterior and segment-polarity integration, but also extend into columnar and temporal integration.

## Material and Methods

### Fly Stocks

The fly stocks used were as follows: *y w exd^B108^ f FRT18D/FM7* [78] and *X*∧*X y f/ovo^D2^ FRT18D/Y;F38/F38* (both provided by R. White). *UAS-exd-GFP* [79] (provided by R. Mann). *exd^1^* [80]. *Antp^25^*and *Antp^14^* [81]. *Antp^Ns-rvC12^* [82]. *UAS-Antp*, *UAS-Ubx*, *UAS-abd-A* [83] (obtained from F. Hirth). *UAS-Abd-Bm* [84] (obtained from J. Castelli-Gair). *Abd-B^M1^* and *Abd-B^M2^* [85]. *Df(3R)Ubx109/Dp(3;3)P5* [86]. *Dp(3;1)P68*; *ss^1^ Ubx^1^ abd-A^D24^ Abd-B^D18^/In(3LR)Ubx^U^*, *Sb^sbd-2^ ss^1^ Ubx^bx-34e^ Ubx^U^* [87]. *C155-Gal4 (elav)* [88]. *abd-A^MX1^* [89]. *Ubx^1^* [90]. *abd-A^P10^* and *Ubx^9.22^* [91]. *hth^5E04^* [92]. *Df(3R)Exel6158* (referred to as *hth^Df3R^*) [93]. *UAS-hth* [94] (provided by A. Salzberg). *col^1^*, *col^3^* [95] and *UAS-col* [96] (provided by A. Vincent). *Df(3L)H99* [97]. *lbe(K)-Gal4* and *UAS-grh* [35]. *ladybird early* fragment K driving *lacZ* (referred to as *lbe(K)-lacZ*) (provided by K. Jagla) [39]. *UAS-nls-myc-EGFP* (referred to as *UAS-nmEGFP*), *UAS-myc-EGFP–farnesylation*, *sqz^Gal4^* [29]. *ap^md544^* (referred to as *ap^Gal4^*) [98]. *ap^rK568^* (referred to as *ap^lacZ^*) [99]. *gsb^01155^* (referred to as *gsb^lacZ^*) [100]. *elav-Gal4* [101] (provided by A. DiAntonio).

Mutants were maintained over *GFP*- or *YFP*-marked balancer chromosomes. As wild type, *w^1118^* was often used. Staging of embryos was performed according to Campos-Ortega and Hartenstein [47]. Unless otherwise stated, flies were obtained from the Bloomington Drosophila Stock Center.

### Immunohistochemistry

Primary antibodies used were: Guinea pig α-Col (1∶1,000), guinea pig α-Dimm (1∶1,000), chicken α-proNplp1 (1∶1,000), and rabbit α-proFMRFa (1∶1,000) [25]. Chicken α-proFMRFa (1∶1,000), chicken α-myc (1∶5,000) and rat α-Grh (1∶1000) [35]. Rabbit α-Nab (1∶1,000) [32] (provided by F. Díaz-Benjumea). Rabbit α-Cas (1∶250) [102] (provided by W. Odenwald). Mouse monoclonal antibody (mAb) α-Col (1∶250) (provided by M. Crozatier and A. Vincent). Guinea pig α-Deadpan (1∶1,000) (provided by J. Skeath). Rat monoclonal α-Gsbn (1∶10) (provided by R. Holmgren). Rabbit α-Hunchback (1∶1,000) and rabbit α-Krüppel (1∶500) (provided by R. Pflanz). mAb α-Nubbin/Pdm1 (1∶10) (provided by S. Cohen). mAb α-Exd (B11M; 1∶5) and mAb α-Ubx (FP3.38; 1∶10) (provided by R. White). mAb α-Abd-A (1∶400) (provided by I. Duncan). Rabbit α-Hth (1∶500) (provided by A. Salzberg). Rabbit α-phospho-histone H3-Ser10 (pH3) (1∶250) and mAb α-myc (1∶2,000). Rabbit α-ß-Gal (1∶5,000). Rabbit α-cleaved caspase-3 (1∶100). Rat monoclonal α-BrdU (1∶100). Chicken α-ß-Gal (1∶1,000). Rabbit α-GFP (1∶500). mAb α-Dac dac2–3 (1∶25), mAb α-Antp (1∶10), mAb α-Abd-B (1∶10), mAb α-Pros MR1A (1∶10), and mAb α-Eya 10H6 (1∶250). All polyclonal sera were preabsorbed against pools of early embryos. Immunostaining was performed according to [35].

### Confocal Imaging and Data Acquisition

Zeiss LSM 5 or Zeiss META 510 confocal microscopes were used to collect data for all fluorescent images; confocal stacks were merged using LSM software or Adobe Photoshop. Where immunolabeling was compared for levels of expression, wild-type and mutant tissue were stained and analyzed on the same slide. Statistical analysis was performed using Microsoft Excel, and bar graphs generated using GraphPad Prism software. Where appropriate, images were false colored to facilitate for color-blind readers.

## Supporting Information

Figure S1 **Expression of Hox and Pbx/Meis factors in the abdominal and thoracic NB 5–6 lineage.** (A and B) Using the NB 5–6 lineage–specific marker *lbe(K)-lacZ*, expression of Antp can be seen at stage 12 in both the thoracic and the abdominal NB 5–6 lineage, whereas expression of Ubx is only observed in the abdominal lineage. (C) Expression of Hth and Exd is found both in the NB 5-6A and NB 5-6T lineages. (D–I) At stage 13, expression of Antp, Ubx, Hth, and Exd is maintained within the NB 5-6A and NB 5-6T lineages and persists into stage 15. At stage 13, Col is specifically expressed in the NB 5-6T lineage. (J–L) At stage 15, using *ap^lacZ^*, expression of Antp, Hth, and Exd is seen in the thoracic Ap-clusters, whereas Ubx is not found in these clusters. Genotypes: (A–I) *lbe(K)-lacZ*. (J–L) *ap^lacZ^*/+.
(9.47 MB TIF)

Figure S2 **Expression of Hox factors in the NB 5–6 lineage throughout the ventral nerve cord.** Determining the anteroposterior extent of Antp, Ubx, abd-A, and Abd-B expression, we find Antp to be expressed within the NB 5–6 lineage from T1 to A9 (A–C), with decreased levels posterior to segment A7. (D and E) Abd-A and Abd-B expression is evident only within the NB 5-6A lineage, spanning A2–A9 (Abd-A) and A7–A9 (Abd-B). Genotypes: (A–E) *lbe(K)-Gal4*, *UAS-nmEGFP*.
(5.18 MB TIF)

Figure S3 **The lineage of abdominal neuroblast 5–6.** (A–G) Expression of Hb, Kr, and Pdm within NB 5-6A at stage 9 to stage early 12. NB 5-6A is identified as the anterior- and lateral-most neuroblast within the *gsb^lacZ^* compartment, or by expression of *lbe(K)-lacZ*, as well as by cell size and staining for Deadpan (unpublished data). Ventral views are shown, with anterior up. (A–H) After NB 5-6A has delaminated, at late stage 8, it coexpresses Hb, Kr, and Pdm (A and E). Hb, Kr, and Pdm are also expressed in a presumable GMC generated by the neuroblast during stage 9 (A′ and E′). At stage 10, expression of Pdm is no longer evident in the neuroblast (B); however, the neuroblast continues to express Hb and Kr through stage 10 (B and F). At stage early 11, expression of Hb is no longer evident within the neuroblast, which is now expressing Kr only (C and G). At stage mid 11, the neuroblast again expresses Pdm (C), and after a short Kr/Pdm coexpression window, Kr is down-regulated and no longer detectable in the neuroblast at stage late 11 (D). (H–M) Staining for Hb, Kr, Pdm, pH3, and cleaved Caspase-3 (Casp-3) within the NB 5-6A lineage in stage 12 embryos. The lineage is visualized using the NB 5–6 lineage–specific reporter construct *lbe(K)-laZ* or *lbe(K)-Gal4*. Images are confocal stacks, subdivided into three or four substacks, from dorsal to ventral (1–2, 1–3, or 1–4). Models are side-view lineage representations deduced from the stacks. Red and green circles depict cells expressing the indicated proteins. White circles depict cells only expressing *lbe(K)-lacZ*. Large circles depict neuroblasts. Semi-large circles indicate presumable GMCs. Dotted lines show substack breakpoints. Midline is to the left, anterior up. (H) At stage early 12, up to five Hb expressing cells can be detected within the NB 5-6A lineage (H1 and H2). One to two of these, usually located at the dorsal end on the lineage, may also express Pdm. These expression data suggest that there are at least three Hb expressing GMCs born in the Hb window between stage late 8 and early 11, one of which is also Pdm expressing. (I) At stage early 12, up to eight cells in the lineage can be found expressing Kr (I1–I3). One or two of the most dorsal Kr cells (I1) and one or two the most ventral Kr cells (I2 and I3) are also expressing Pdm at this stage, suggesting that there is at least one GMC generated in the Kr/Pdm coexpression window during stage 9, three Kr-only GMCs generated during stages 10 to 11, and yet another Kr/Pdm coexpressing GMC generated during stage late 11, after which the neuroblast does no longer express Kr. Most ventral within the lineage (H2 and I3), a Pdm-only expressing semi-large cell can be detected at stage early 12, temporally coinciding with the last pH3 activity seen in the lineage (see [J]), indicating that the last GMC born from 5-6A is a Pdm-only GMC. (J) Stage early 12 is the last stage in which pH3 staining can be detected within the 5-6A lineage. Typical postmitotic pH3 staining can be seen both within the NB (J3), and a more dorsal semi-large cell (J2; a presumable NB→NB/GMC division), as well as in a pair of smaller cells more dorsally within the lineage (J1 and J2; a presumable GMC→neurons/glia division). (K) At stage late 12 and onward, no pH3 staining is detectable within the 5-6A lineage. (L and M) Staining for Casp-3 reveals that several cells within the 5-6A lineage, at dorsal, intermediate, and ventral positions, undergo apoptosis during stage late 12. (N) Model showing the progression of temporal gene expression within the NB 5-6A lineage. NB 5-6A generates 12 neurons/glia between stage 9 and early 12, after which it exits the cell cycle and presumably dies. Genotypes: (A–H) *gsb^lacZ^*/+. (I–M) *lbe(K)-lacZ*. (N) *lbe(K)-Gal4*, *UAS-nmGFP*.
(4.14 MB TIF)

Figure S4 **Suppression of thoracic NB 5–6 lineage by Ubx and Pbx/Meis factors.** (A and B) Control and *Ubx* misexpression, stage 15, using *lbe(K)-Gal4*. *Ubx* triggers a smaller NB 5-6T lineage, and reduced or absent expression of Cas and Col. (C) Quantification of GFP, Cas, and Col expressing cells/NB 5-6T lineage, at stage 15 control and *Ubx* misexpression VNCs (*n*>20 lineages). Asterisks denote significant difference compared to thoracic control (*p*<0.01, Student two-tailed test). (D and E) Postmitotic misexpression of *Ubx* from *ap^Gal4^* does not disrupt Ap cluster differentiation, as evident by expression of Eya, Dimm, Nplp1, and FMRFa. (F) Staining for Ubx reveals that Ubx is expressed at high levels in all four Ap neurons using this driver. Genotypes: (A) *lbe(K)-Gal4*, *UAS-nmEGFP/+*; *lbe(K)-Gal4*, *UAS-nmEGFP*/+. (B) *lbe(K)-Gal4,UAS-nmEGFP;Ubx*. (C) Genotypes as in (A and B). (D) *w^1118^*. (E) *ap^Gal4^/UAS-Ubx*. (F) *ap^Gal4^*, *UAS-nmEGFP/UAS-Ubx*.
(1.70 MB TIF)

Figure S5 ***Antp*, hth, and *exd* play critical roles during Ap cluster specification.** (A–D) Expression of the two neuropeptides, FMRFa and Nplp1, in *w^1118^*, *Antp*, *hth*, and *exd* mutant VNCs, at stage 18 h AEL. Expression of both Nplp1 and FMRFa is completely lost in the Ap clusters (bracket). Nplp1 expression is still apparent in dorsal Ap neurons in all three mutants, and FMRFa in the anterior SE2 neurons. (E–H) Expression of Eya and *ap^Gal4^* in control, *Antp*, *hth*, and *exd* mutant thoracic segment, at stage 15 (hatched line marks the midline). Expression of Eya and *ap* is completely lost or strongly reduced in all three mutant backgrounds. (I–L) Expression of Nab in control, *Antp*, *hth*, and *exd* stage 15 thoracic segments reveals no effect upon Nab expression within the NB 5-6T lineage. (M–P) Expression of Col in control, *Antp*, *hth*, and *exd* stage 14 thoracic segments reveals loss of Col in *Antp*, and strong reduction of Col expression in *hth* and *exd*. (Q–T) Expression of *sqz^Gal4^*, Dac, and Dimm in control, *Antp*, *hth*, and *exd* mutant stage 16 thoracic segments. In all three mutant backgrounds, Dimm and Dac expression is lost when compared to wild type, whereas *sqz^Gal4^* expression is unaffected. (U) Quantification of thoracic, lateral cells/VNC expressing FMRFa and Nplp1 (*n*>7 VNCs). (V) Quantification of Eya and *ap^Gal4^* positive cells/Ap cluster in T2/T3 thoracic segments (*n*>11 VNCs). (X) Quantification of Nab-positive cells/NB 5-6T lineage (*n*>12 lineages). (Y) Quantification of Col-positive cells/NB 5-6T lineage (*n*>8 lineages). (Z) Quantification of *sqz^Gal4^*, Dac and Dimm positive cells/NB 5-6T lineage (*n*>11 lineages). Asterisks denote significant difference compared to control (*p*<0.01, Student two-tailed test). *exd* is maternally provided, but the less severe phenotypes in *exd* does not result from compensating maternal load, since we were analyzing embryos mutant both for maternal and zygotic *exd* function. Genotypes: (A) *w^1118^*. (B) *Antp^25^/Antp^Ns-rvC12^*. (C) *hth^5E04^/hth^Df3R^*. (D) *exd^B108^*, *FRT^18D^/y*. (E) *ap^Gal4^/UAS-nmEGFP*. (F) *ap^Gal4^/UAS-nmEGFP*; *Antp^25^/Antp^Ns-rvC12^*. (G) *ap^Gal4^/UAS-nmEGFP*; *hth^5E04^/hth^Df3R^*. (H) *exd^B108^*, *FRT^18D^/y*; *ap^Gal4^*, *UAS-nmEGFP/+*. (I) *lbe(K)-Gal4*, *UAS-nmEGFP/+*; *lbe(K)-Gal4*, *UAS-nmEGFP/+*. (J) *lbe(K)-Gal4*, *UAS-nmEGFP*; *Antp^25^/Antp^Ns-rvC12^*. (K) *lbe(K)-Gal4*, *UAS-nmEGFP*; *hth^5E04^/hth^Df3R^*. (L) *exd^1^*/y; *lbe(K)-Gal4*, *UAS-nmEGFP*/+. (M) *UAS-nmEGFP/+*; *lbe(K)-Gal4/+*. (N) *lbe(K)-Gal4*, *UAS-nmEGFP/+*; *Antp^25^/Antp^Ns-rvC12^*. (O) *lbe(K)-lacZ*; *hth^5E04^/hth^Df3R^*. (P) *exd^B108^*, *FRT^18D^/y*; *lbe(K)-Gal4*, *UAS-nmEGFP*/+; *lbe(K)-Gal4*, *UAS-nmEGFP*/+. (Q) *lbe(K)-lacZ*, *UAS-nmEGFP*/*lbe(K)-lacZ;sqz^Gal4^*/+. (R) *lbe(K)-lacZ*, *UAS-nmGFP*; *Antp^25^*,*sqz^Gal4^*/*Antp^Ns-rvC12^*. (S) *lbe(K)-lacZ*, *UAS-nmEGFP*; *hth^5E04^*,*sqz^Gal4^*/*hth^Df3R^*. (T) *exd^B108^*, *FRT^18D^/y*;;*sqz^Gal4^,UAS-nmEGFP*/+.
(2.53 MB TIF)

Figure S6 **Expression of *lbe(K)-Gal4* and *elav-Gal4* in the abdominal NB 5–6 lineage.** Expression of *UAS-nmEGFP*, driven from *lbe(K)-Gal4* and *elav-Gal4*, and detected with anti-Myc/GFP expression. Abdominal row 5, lateral compartment is identified by expression of Gsbn. Expression of the two drivers commences at different time points. (A–C) In contrast to control and *elav>nmEGFP*, *lbe(K)>nmEGFP* expression is observed at stage 11. (D–I) Expression from both drivers can be observed at stage 12 (D–F), and into stage 13 (G–I). All genotypes were processed on the same slide and scanned using identical confocal settings. Genotypes: (A, D, and G) *w^1118^*. (B, E, and H) *lbe(K)-Gal4/UAS-nmEGFP*. (C, F, and I) *UAS-nmEGFP/+*; *elav-Gal4/+*.
(8.89 MB TIF)

Figure S7 **Segment-specific modifications of the NB 5–6 lineage in anterior segments, with respect to lineage size and temporal gene expression.** (A) Expression of Cas, Grh, and Col in anterior NB 5–6 lineages. Images are from embryos processed on the same slide, using identical confocal settings. (B) Quantification of lineage size (black bars), Cas (red), Grh (yellow), and Col expression (blue) in anterior NB 5–6 lineages (cells/lineage; *n*>10). (C) Cartoon summarizing the analysis of the NB 5–6 lineage in the brain and subesophageal segments. There are segment-specific modifications of the NB 5–6 lineage, both with respect to lineage size and gene expression. Most pertinently, although there is no expression of Col above segment S2, Cas is expressed in all anterior NB 5–6 lineages, and all segments but S1 show some level of Grh expression. Genotypes: *lbe(K)-Gal4*, *UAS-GFP/+*; *lbe(K)-Gal4*, *UAS-GFP/+*.
(0.97 MB TIF)

Figure S8 ***Antp* misexpression in anterior NB 5–6 triggers Ap cluster formation.** (A and B) Misexpression of *Antp* in anterior NB 5–6 lineages, from *lbe(K)-Gal4*, triggers ectopic Ap cluster specification, evident from expression of Eya and Nplp1, here exemplified in segment B3. (C and D) Misexpression of *Antp* and *grh* in anterior NB 5–6 lineages, from *lbe(K)-Gal4*, triggers more complete ectopic Ap cluster specification, evident from expression of Eya and FMRFa, here exemplified in segment B3. Genotypes: (A and C) *lbe(K)-Gal4,UAS-nmEGFP/+*. (B) *lbe(K)-Gal4,UAS-nmEGFP/UAS-Antp*. (D) *lbe(K)-Gal4,UAS-nmEGFP/UAS-Antp*, *UAS-grh*.
(3.13 MB TIF)

Figure S9 **Within thoracic NB 5–6, Hox and Pbx/Meis genes do not regulate temporal genes, and vice versa.** (A–D and K) Expression of Cas and Grh in control, *Antp*, *hth*, and *exd* mutants, reveals no effects upon expression. (E–J and L) Expression of Antp, Hth, and Exd in control, *cas* and *grh* mutant background reveals no effects upon expression. Hatched bar marks midline; one thoracic, stage 16, segment. (K and L) Quantification of the total number of cells/lineage expressing GFP, Cas, Grh, Antp, Hth, and Exd (cells/lineage; *n*>11 lineages). Although gene expression is not affected, we find that *Antp* and *cas* mutants have additional cells in the NB 5-6T lineage. Asterisks denotes significant difference compared to control (*p*<0.01; Student two-tailed test). Genotypes: (A, E, and H) *lbe(K)-Gal4*, *UAS-nmEGFP/+*; *lbe(K)-Gal4*, *UAS-nmEGFP/+*. (B, F, and I) *lbe(K)-Gal4*, *UAS-nmEGFP/+*; *Antp^25^/Antp^Ns-rvC12^*. (C, G, and J) *lbe(K)-Gal4*, *UAS-nmEGFP*; *hth^5E04^/hth^Df3R^*. (D) *exd^1^/y*; *lbe(K)-Gal4*, *UAS-nmEGFP/+*; *lbe(K)-Gal4*, *UAS-nmEGFP/+*.
(3.75 MB TIF)

Figure S10 ***Antp* misexpression does not lead to homeotic transformation of anterior NB 5–6 lineages.** Quantification of the number of cells expressing GFP, Cas, Grh, and Col in the anterior NB 5–6 lineages, in control (top) and *Antp* misexpression (bottom), at stage 15 (cells/lineage; *n*>12 lineages). Whereas Col is ectopically activated by Antp, there are no significant changes in NB 5–6 lineage cell numbers, nor in Cas or Grh cell numbers. Asterisks denotes significant difference compared to control (*p*<0.01; Student two-tailed test). Genotypes: (A) *lbe(K)-Gal4*, *UAS-nmEGFP/+*, (B) *lbe(K)-Gal4*, *UAS-nmEGFP/UAS-Antp*.
(0.21 MB TIF)

## Abbreviations

AEL: after egg laying
Bx-C: bithorax complex
CNS: central nervous system
GMC: ganglion mother cell
VNC: ventral nerve cord
