# Supplementary figures and images for: Segment-Specific Neuronal Subtype Specification by the Integration of Anteroposterior and Temporal Cues
Source: PLoS Biol. 2010 May 11;8(5):e1000368. doi: 10.1371/journal.pbio.1000368 (PMC2867937; doi:10.1371/journal.pbio.1000368)

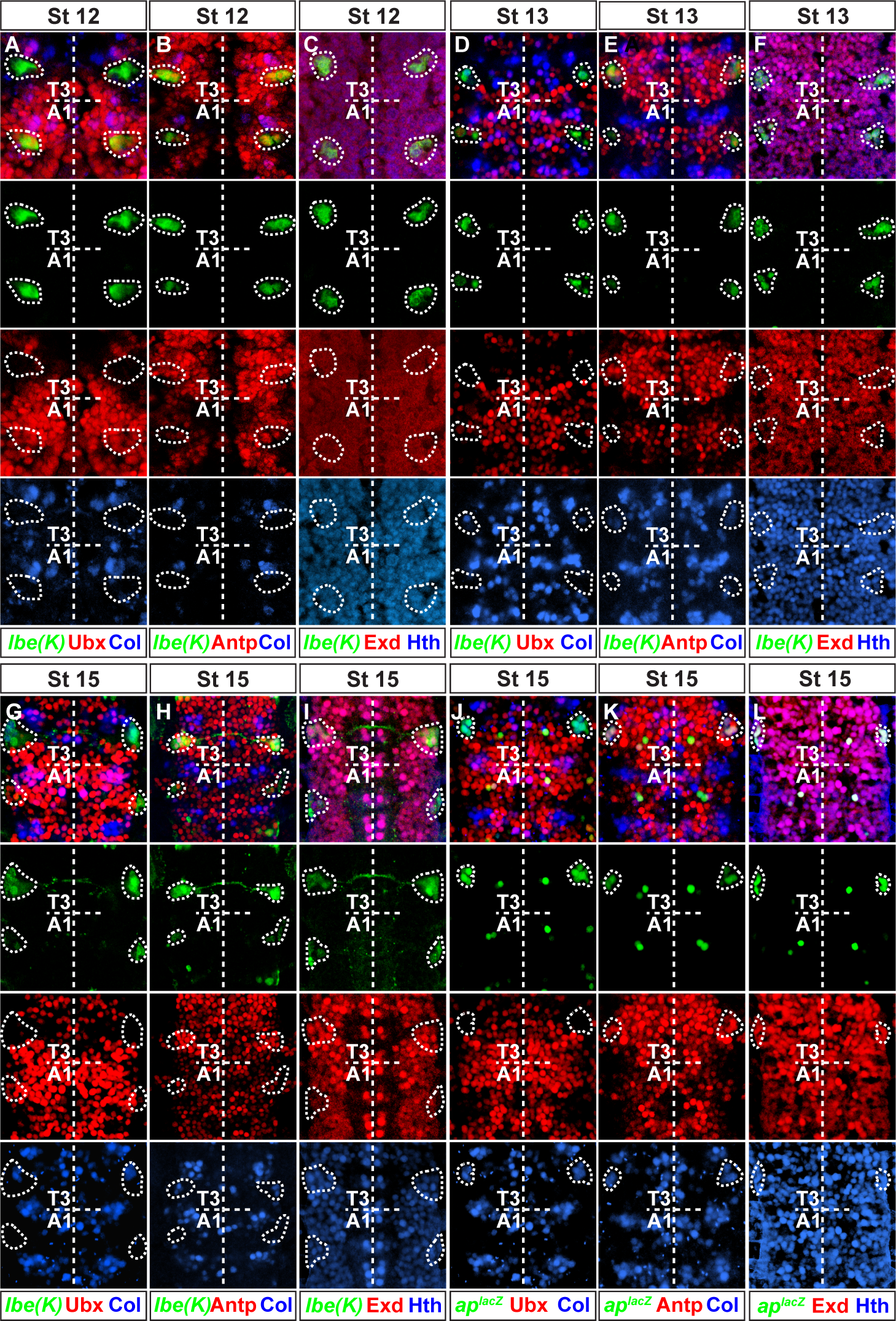

Supplement: Figure S1 — Expression of Hox and Pbx/Meis factors in the abdominal and thoracic NB 5–6 lineage. (A and B) Using the NB 5–6 lineage–specific marker lbe(K)-lacZ, expression of Antp can be seen at stage 12 in both the thoracic and the abdominal NB 5–6 lineage, whereas expression of Ubx is only observed in the abdominal lineage. (C) Expression of Hth and Exd is found both in the NB 5-6A and NB 5-6T lineages. (D–I) At stage 13, expression of Antp, Ubx, Hth, and Exd is maintained within the NB 5-6A and NB 5-6T lineages and persists into stage 15. At stage 13, Col is specifically expressed in the NB 5-6T lineage. (J–L) At stage 15, using aplacZ, expression of Antp, Hth, and Exd is seen in the thoracic Ap-clusters, whereas Ubx is not found in these clusters. Genotypes: (A–I) lbe(K)-lacZ. (J–L) aplacZ/+. (9.47 MB TIF) [file pbio.1000368.s001.tif]

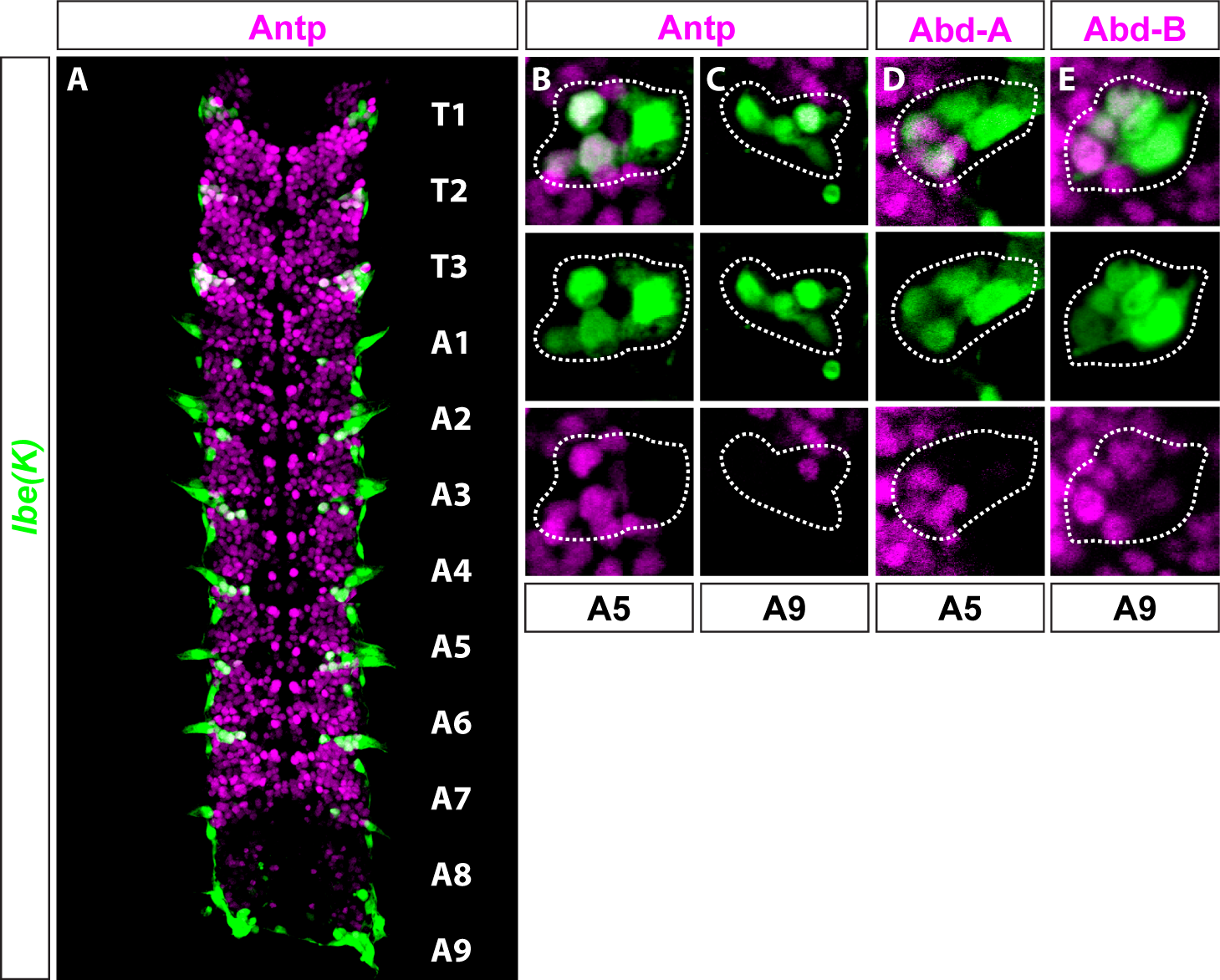

Supplement: Figure S2 — Expression of Hox factors in the NB 5–6 lineage throughout the ventral nerve cord. Determining the anteroposterior extent of Antp, Ubx, abd-A, and Abd-B expression, we find Antp to be expressed within the NB 5–6 lineage from T1 to A9 (A–C), with decreased levels posterior to segment A7. (D and E) Abd-A and Abd-B expression is evident only within the NB 5-6A lineage, spanning A2–A9 (Abd-A) and A7–A9 (Abd-B). Genotypes: (A–E) lbe(K)-Gal4, UAS-nmEGFP. (5.18 MB TIF) [file pbio.1000368.s002.tif]

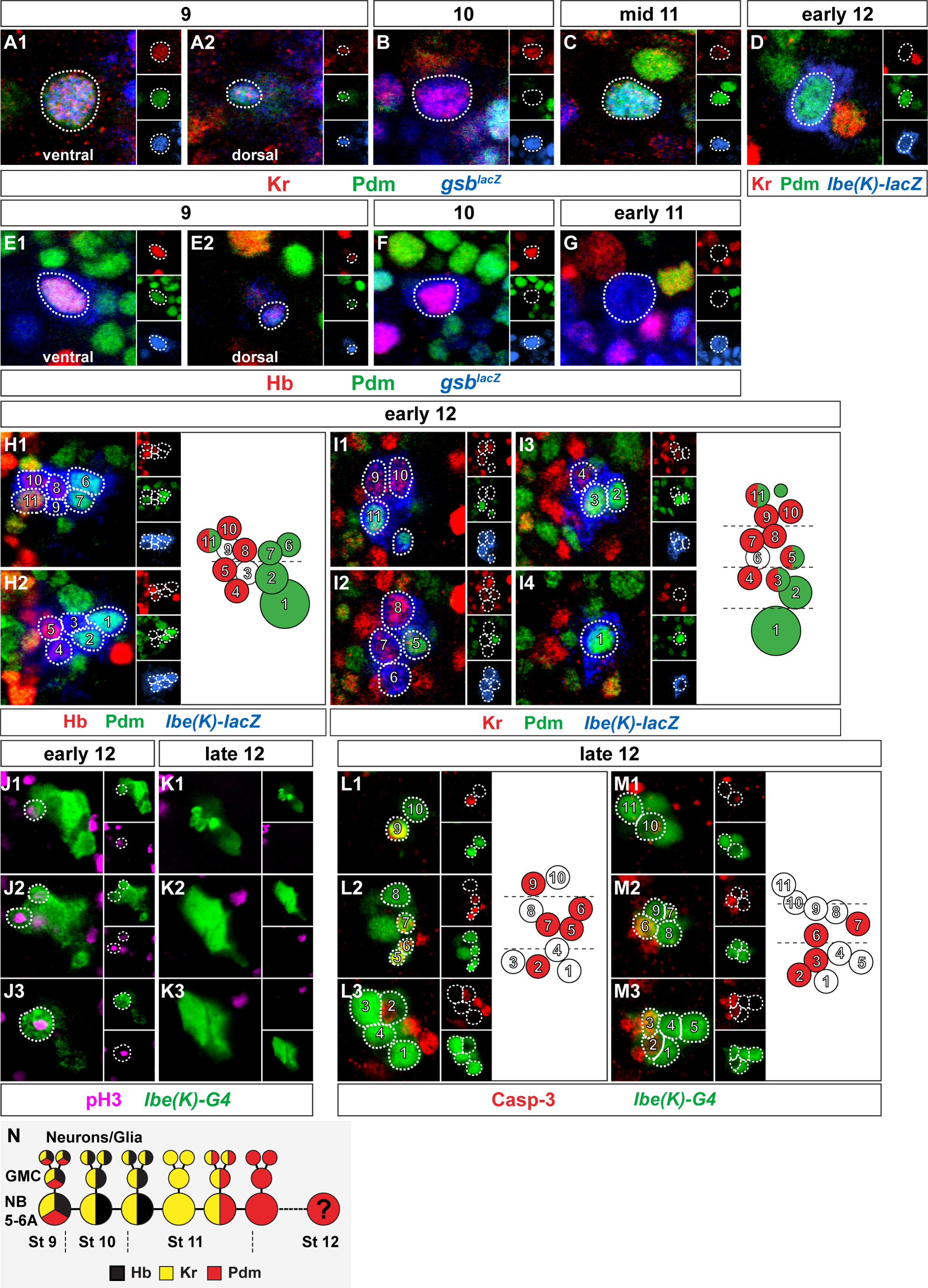

Supplement: Figure S3 — The lineage of abdominal neuroblast 5–6. (A–G) Expression of Hb, Kr, and Pdm within NB 5-6A at stage 9 to stage early 12. NB 5-6A is identified as the anterior- and lateral-most neuroblast within the gsblacZ compartment, or by expression of lbe(K)-lacZ, as well as by cell size and staining for Deadpan (unpublished data). Ventral views are shown, with anterior up. (A–H) After NB 5-6A has delaminated, at late stage 8, it coexpresses Hb, Kr, and Pdm (A and E). Hb, Kr, and Pdm are also expressed in a presumable GMC generated by the neuroblast during stage 9 (A′ and E′). At stage 10, expression of Pdm is no longer evident in the neuroblast (B); however, the neuroblast continues to express Hb and Kr through stage 10 (B and F). At stage early 11, expression of Hb is no longer evident within the neuroblast, which is now expressing Kr only (C and G). At stage mid 11, the neuroblast again expresses Pdm (C), and after a short Kr/Pdm coexpression window, Kr is down-regulated and no longer detectable in the neuroblast at stage late 11 (D). (H–M) Staining for Hb, Kr, Pdm, pH3, and cleaved Caspase-3 (Casp-3) within the NB 5-6A lineage in stage 12 embryos. The lineage is visualized using the NB 5–6 lineage–specific reporter construct lbe(K)-laZ or lbe(K)-Gal4. Images are confocal stacks, subdivided into three or four substacks, from dorsal to ventral (1–2, 1–3, or 1–4). Models are side-view lineage representations deduced from the stacks. Red and green circles depict cells expressing the indicated proteins. White circles depict cells only expressing lbe(K)-lacZ. Large circles depict neuroblasts. Semi-large circles indicate presumable GMCs. Dotted lines show substack breakpoints. Midline is to the left, anterior up. (H) At stage early 12, up to five Hb expressing cells can be detected within the NB 5-6A lineage (H1 and H2). One to two of these, usually located at the dorsal end on the lineage, may also express Pdm. These expression data suggest that there are at least three Hb expre [file pbio.1000368.s003.tif]

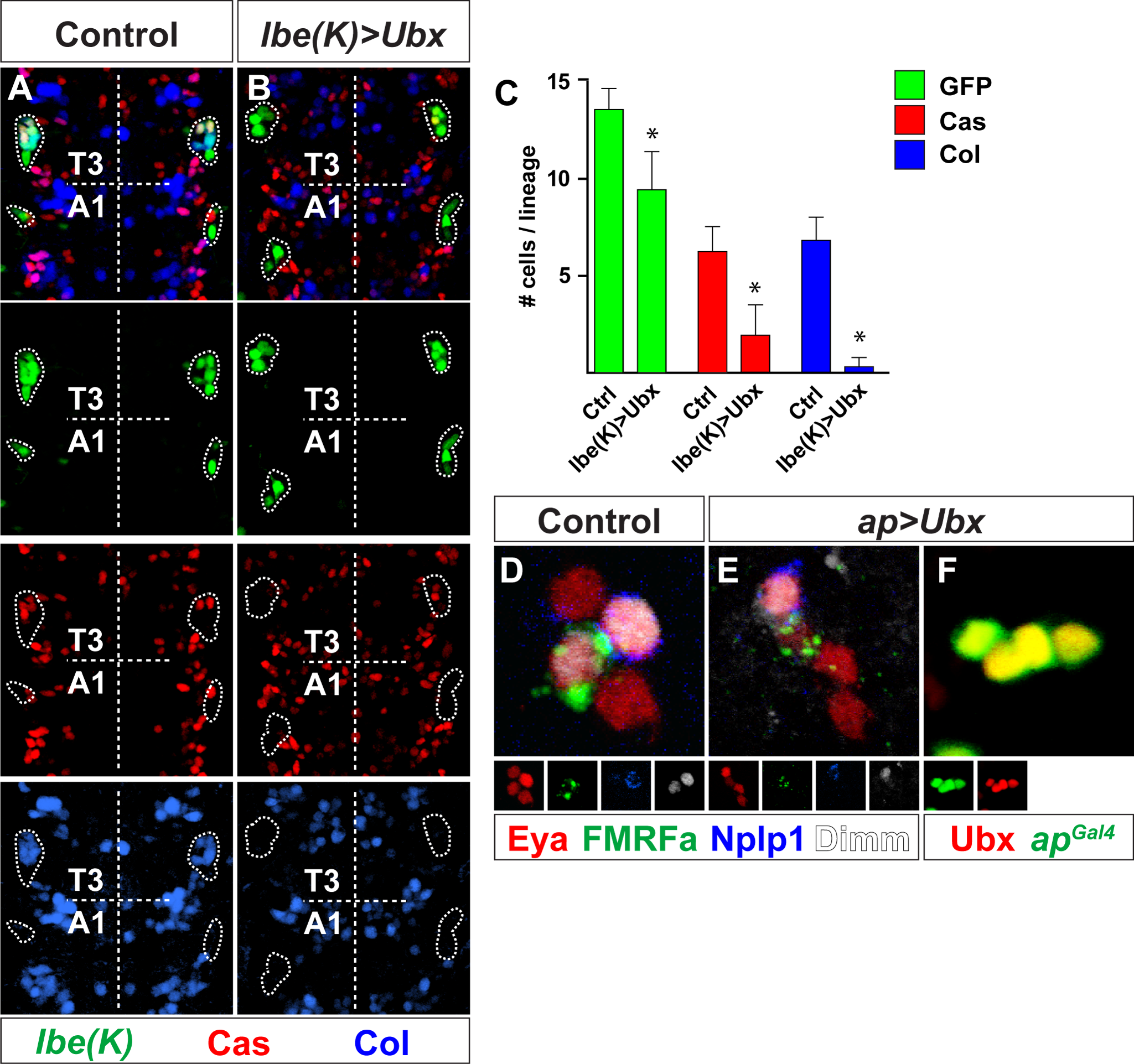

Supplement: Figure S4 — Suppression of thoracic NB 5–6 lineage by Ubx and Pbx/Meis factors. (A and B) Control and Ubx misexpression, stage 15, using lbe(K)-Gal4. Ubx triggers a smaller NB 5-6T lineage, and reduced or absent expression of Cas and Col. (C) Quantification of GFP, Cas, and Col expressing cells/NB 5-6T lineage, at stage 15 control and Ubx misexpression VNCs (n>20 lineages). Asterisks denote significant difference compared to thoracic control (p<0.01, Student two-tailed test). (D and E) Postmitotic misexpression of Ubx from apGal4 does not disrupt Ap cluster differentiation, as evident by expression of Eya, Dimm, Nplp1, and FMRFa. (F) Staining for Ubx reveals that Ubx is expressed at high levels in all four Ap neurons using this driver. Genotypes: (A) lbe(K)-Gal4, UAS-nmEGFP/+; lbe(K)-Gal4, UAS-nmEGFP/+. (B) lbe(K)-Gal4,UAS-nmEGFP;Ubx. (C) Genotypes as in (A and B). (D) w1118. (E) apGal4/UAS-Ubx. (F) apGal4, UAS-nmEGFP/UAS-Ubx. (1.70 MB TIF) [file pbio.1000368.s004.tif]

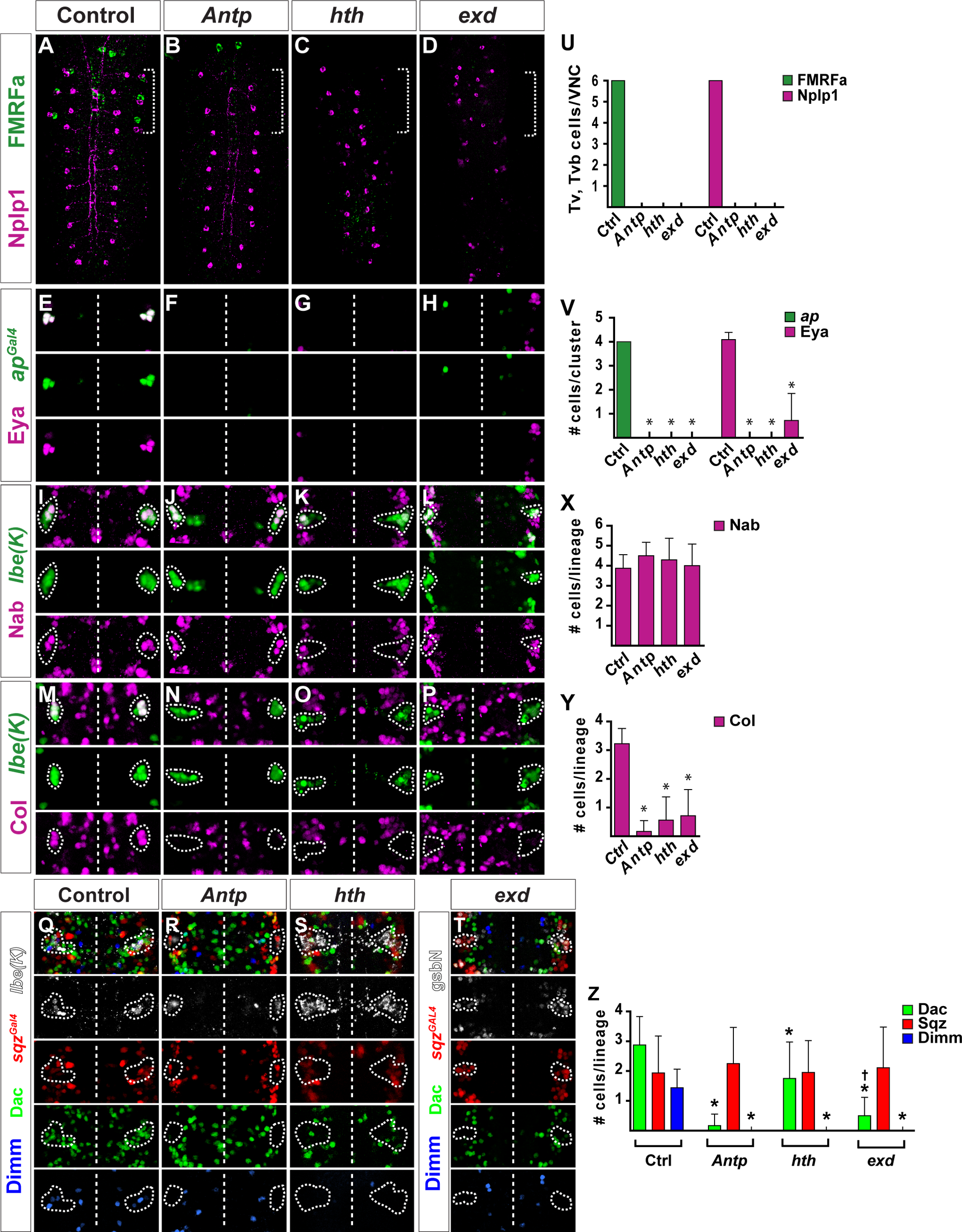

Supplement: Figure S5 — Antp, hth, and exd play critical roles during Ap cluster specification. (A–D) Expression of the two neuropeptides, FMRFa and Nplp1, in w1118, Antp, hth, and exd mutant VNCs, at stage 18 h AEL. Expression of both Nplp1 and FMRFa is completely lost in the Ap clusters (bracket). Nplp1 expression is still apparent in dorsal Ap neurons in all three mutants, and FMRFa in the anterior SE2 neurons. (E–H) Expression of Eya and apGal4 in control, Antp, hth, and exd mutant thoracic segment, at stage 15 (hatched line marks the midline). Expression of Eya and ap is completely lost or strongly reduced in all three mutant backgrounds. (I–L) Expression of Nab in control, Antp, hth, and exd stage 15 thoracic segments reveals no effect upon Nab expression within the NB 5-6T lineage. (M–P) Expression of Col in control, Antp, hth, and exd stage 14 thoracic segments reveals loss of Col in Antp, and strong reduction of Col expression in hth and exd. (Q–T) Expression of sqzGal4, Dac, and Dimm in control, Antp, hth, and exd mutant stage 16 thoracic segments. In all three mutant backgrounds, Dimm and Dac expression is lost when compared to wild type, whereas sqzGal4 expression is unaffected. (U) Quantification of thoracic, lateral cells/VNC expressing FMRFa and Nplp1 (n>7 VNCs). (V) Quantification of Eya and apGal4 positive cells/Ap cluster in T2/T3 thoracic segments (n>11 VNCs). (X) Quantification of Nab-positive cells/NB 5-6T lineage (n>12 lineages). (Y) Quantification of Col-positive cells/NB 5-6T lineage (n>8 lineages). (Z) Quantification of sqzGal4, Dac and Dimm positive cells/NB 5-6T lineage (n>11 lineages). Asterisks denote significant difference compared to control (p<0.01, Student two-tailed test). exd is maternally provided, but the less severe phenotypes in exd does not result from compensating maternal load, since we were analyzing embryos mutant both for maternal and zygotic exd function. Genotypes: (A) w1118. (B) Antp25/AntpNs-rvC12. (C) hth5E04/hthDf3R. (D) exdB108, FRT18D/y. [file pbio.1000368.s005.tif]

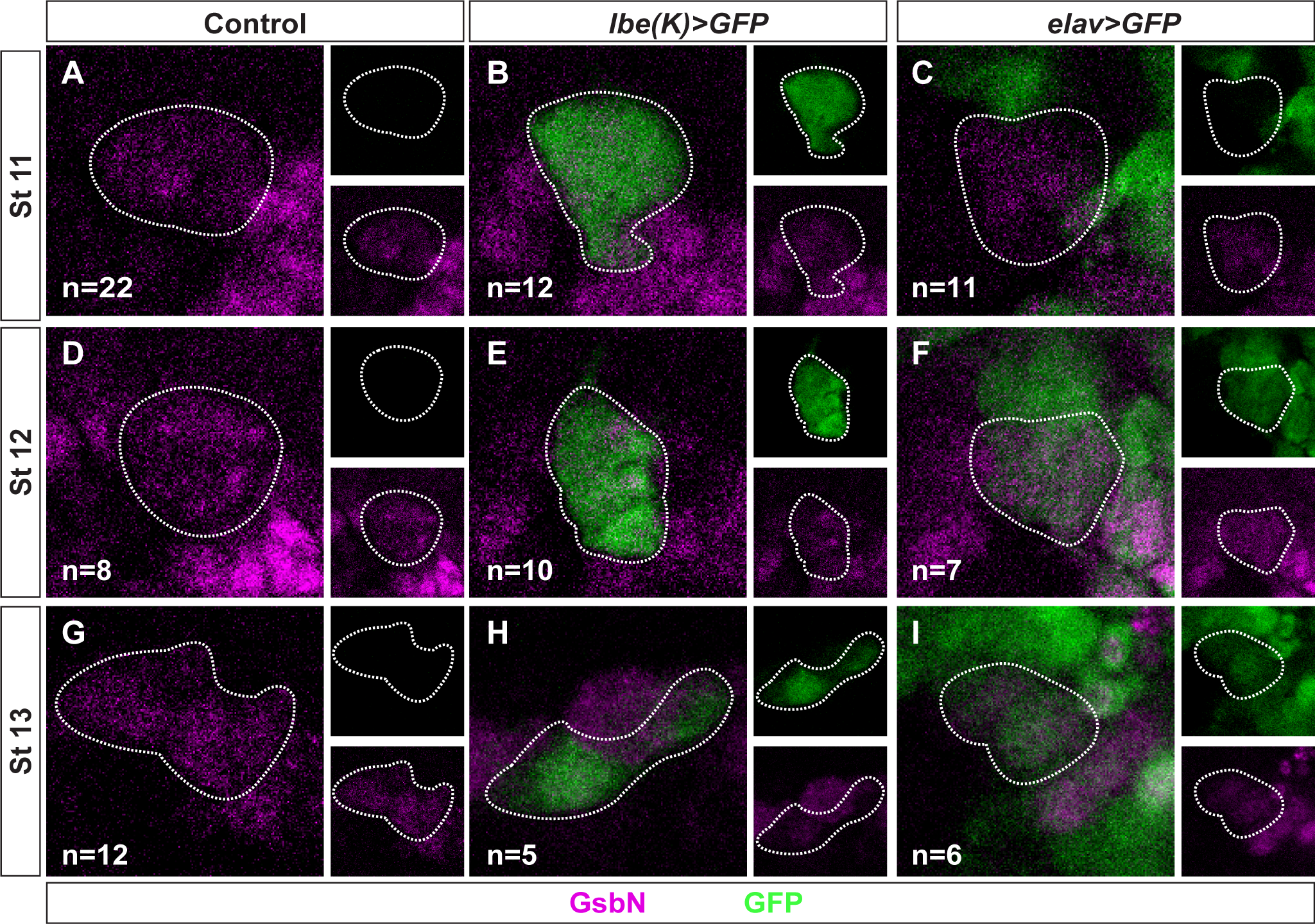

Supplement: Figure S6 — Expression of lbe(K)-Gal4 and elav-Gal4 in the abdominal NB 5–6 lineage. Expression of UAS-nmEGFP, driven from lbe(K)-Gal4 and elav-Gal4, and detected with anti-Myc/GFP expression. Abdominal row 5, lateral compartment is identified by expression of Gsbn. Expression of the two drivers commences at different time points. (A–C) In contrast to control and elav>nmEGFP, lbe(K)>nmEGFP expression is observed at stage 11. (D–I) Expression from both drivers can be observed at stage 12 (D–F), and into stage 13 (G–I). All genotypes were processed on the same slide and scanned using identical confocal settings. Genotypes: (A, D, and G) w1118. (B, E, and H) lbe(K)-Gal4/UAS-nmEGFP. (C, F, and I) UAS-nmEGFP/+; elav-Gal4/+. (8.89 MB TIF) [file pbio.1000368.s006.tif]

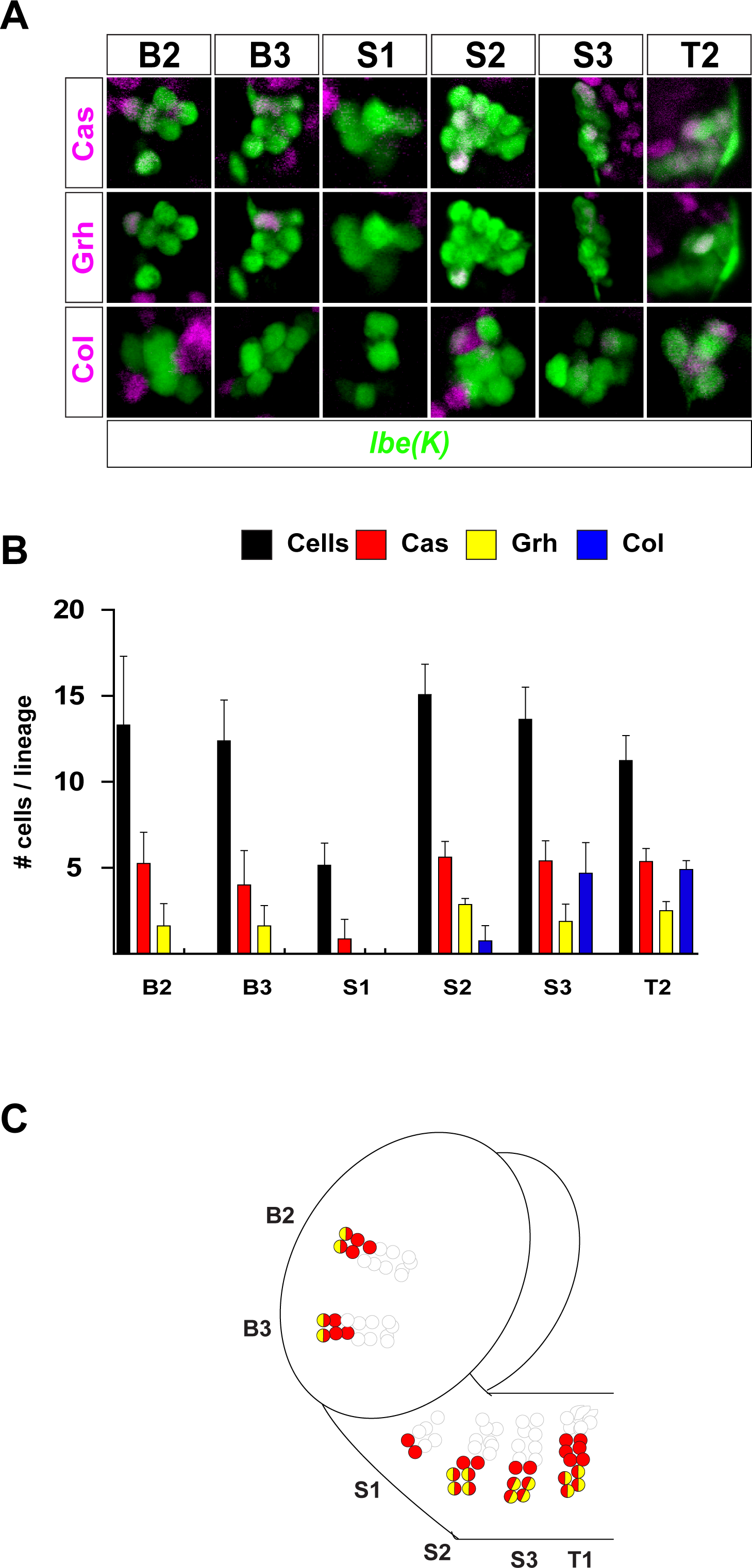

Supplement: Figure S7 — Segment-specific modifications of the NB 5–6 lineage in anterior segments, with respect to lineage size and temporal gene expression. (A) Expression of Cas, Grh, and Col in anterior NB 5–6 lineages. Images are from embryos processed on the same slide, using identical confocal settings. (B) Quantification of lineage size (black bars), Cas (red), Grh (yellow), and Col expression (blue) in anterior NB 5–6 lineages (cells/lineage; n>10). (C) Cartoon summarizing the analysis of the NB 5–6 lineage in the brain and subesophageal segments. There are segment-specific modifications of the NB 5–6 lineage, both with respect to lineage size and gene expression. Most pertinently, although there is no expression of Col above segment S2, Cas is expressed in all anterior NB 5–6 lineages, and all segments but S1 show some level of Grh expression. Genotypes: lbe(K)-Gal4, UAS-GFP/+; lbe(K)-Gal4, UAS-GFP/+. (0.97 MB TIF) [file pbio.1000368.s007.tif]

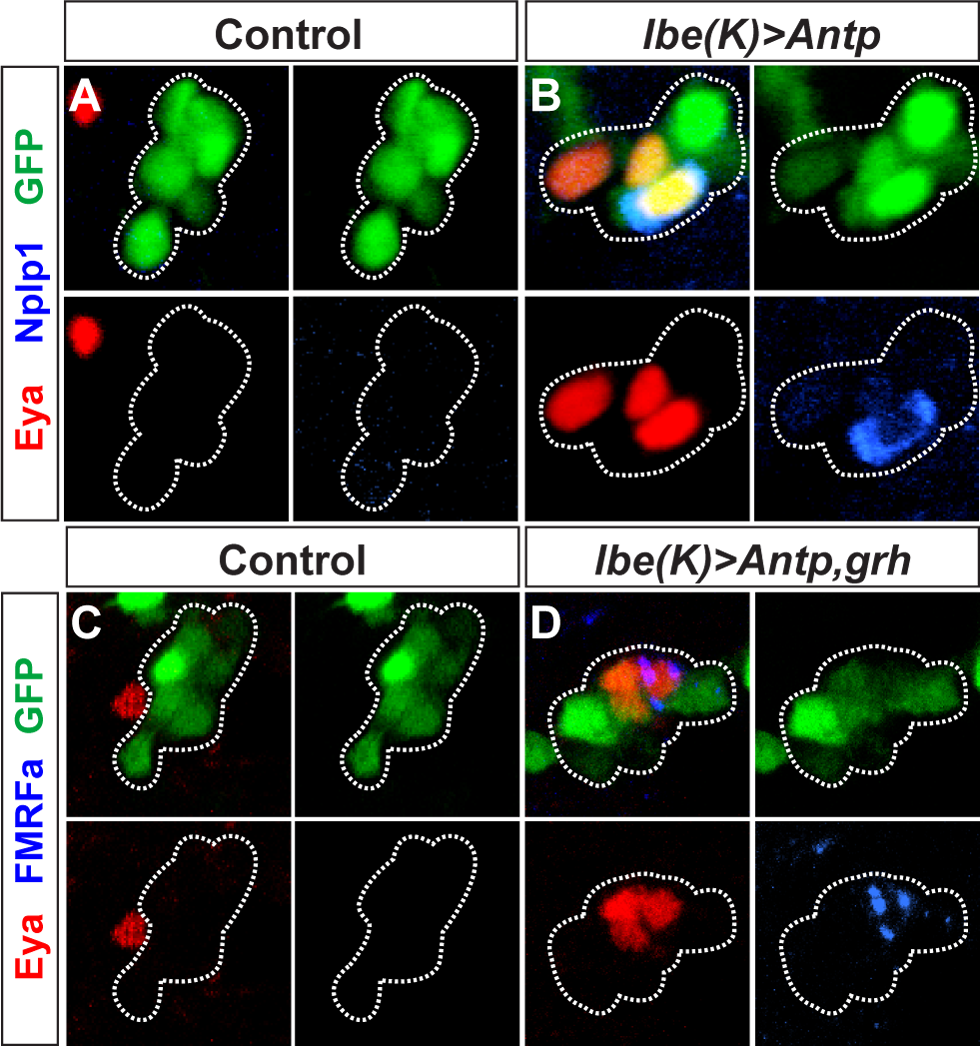

Supplement: Figure S8 — Antp misexpression in anterior NB 5–6 triggers Ap cluster formation. (A and B) Misexpression of Antp in anterior NB 5–6 lineages, from lbe(K)-Gal4, triggers ectopic Ap cluster specification, evident from expression of Eya and Nplp1, here exemplified in segment B3. (C and D) Misexpression of Antp and grh in anterior NB 5–6 lineages, from lbe(K)-Gal4, triggers more complete ectopic Ap cluster specification, evident from expression of Eya and FMRFa, here exemplified in segment B3. Genotypes: (A and C) lbe(K)-Gal4,UAS-nmEGFP/+. (B) lbe(K)-Gal4,UAS-nmEGFP/UAS-Antp. (D) lbe(K)-Gal4,UAS-nmEGFP/UAS-Antp, UAS-grh. (3.13 MB TIF) [file pbio.1000368.s008.tif]

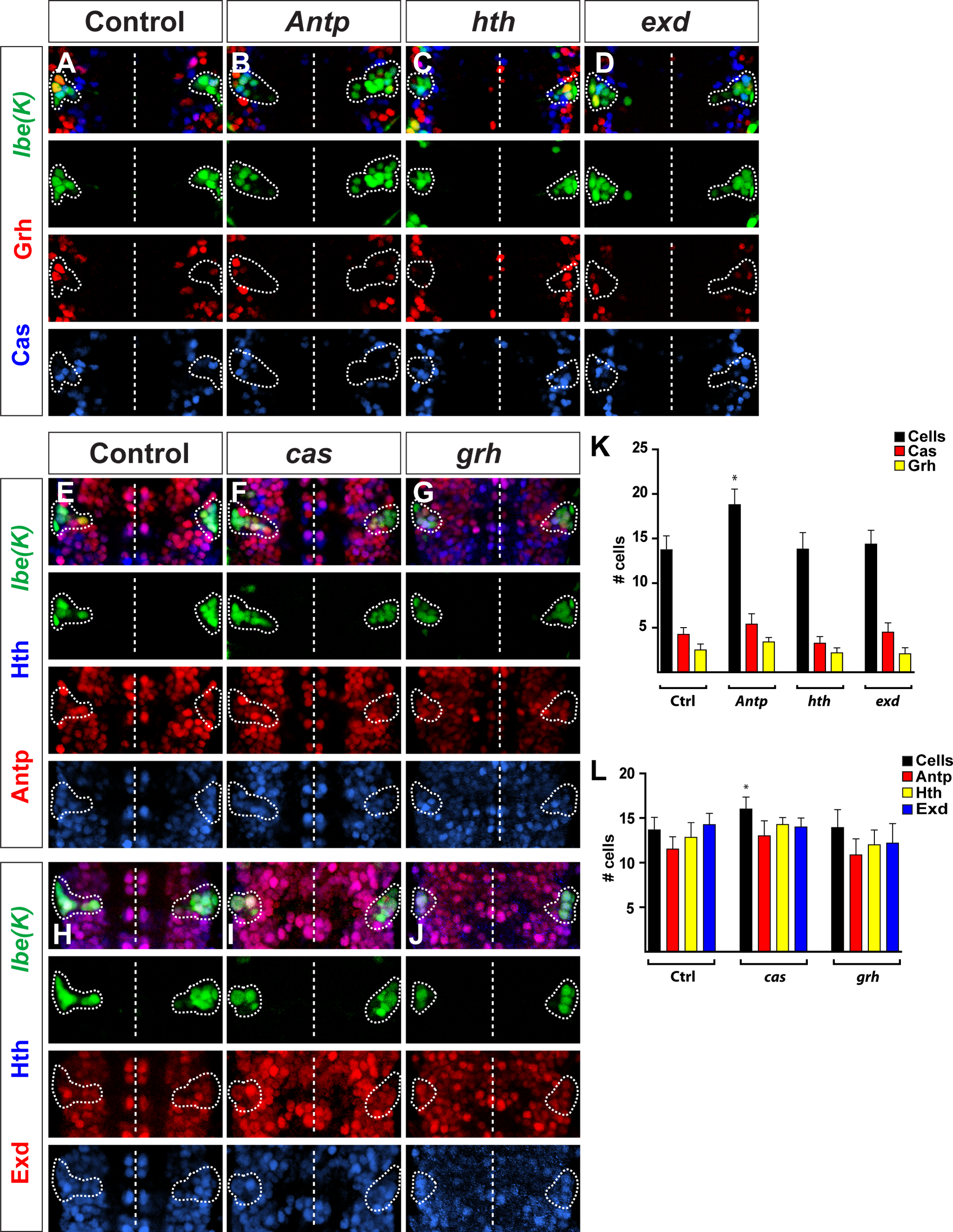

Supplement: Figure S9 — Within thoracic NB 5–6, Hox and Pbx/Meis genes do not regulate temporal genes, and vice versa. (A–D and K) Expression of Cas and Grh in control, Antp, hth, and exd mutants, reveals no effects upon expression. (E–J and L) Expression of Antp, Hth, and Exd in control, cas and grh mutant background reveals no effects upon expression. Hatched bar marks midline; one thoracic, stage 16, segment. (K and L) Quantification of the total number of cells/lineage expressing GFP, Cas, Grh, Antp, Hth, and Exd (cells/lineage; n>11 lineages). Although gene expression is not affected, we find that Antp and cas mutants have additional cells in the NB 5-6T lineage. Asterisks denotes significant difference compared to control (p<0.01; Student two-tailed test). Genotypes: (A, E, and H) lbe(K)-Gal4, UAS-nmEGFP/+; lbe(K)-Gal4, UAS-nmEGFP/+. (B, F, and I) lbe(K)-Gal4, UAS-nmEGFP/+; Antp25/AntpNs-rvC12. (C, G, and J) lbe(K)-Gal4, UAS-nmEGFP; hth5E04/hthDf3R. (D) exd1/y; lbe(K)-Gal4, UAS-nmEGFP/+; lbe(K)-Gal4, UAS-nmEGFP/+. (3.75 MB TIF) [file pbio.1000368.s009.tif]

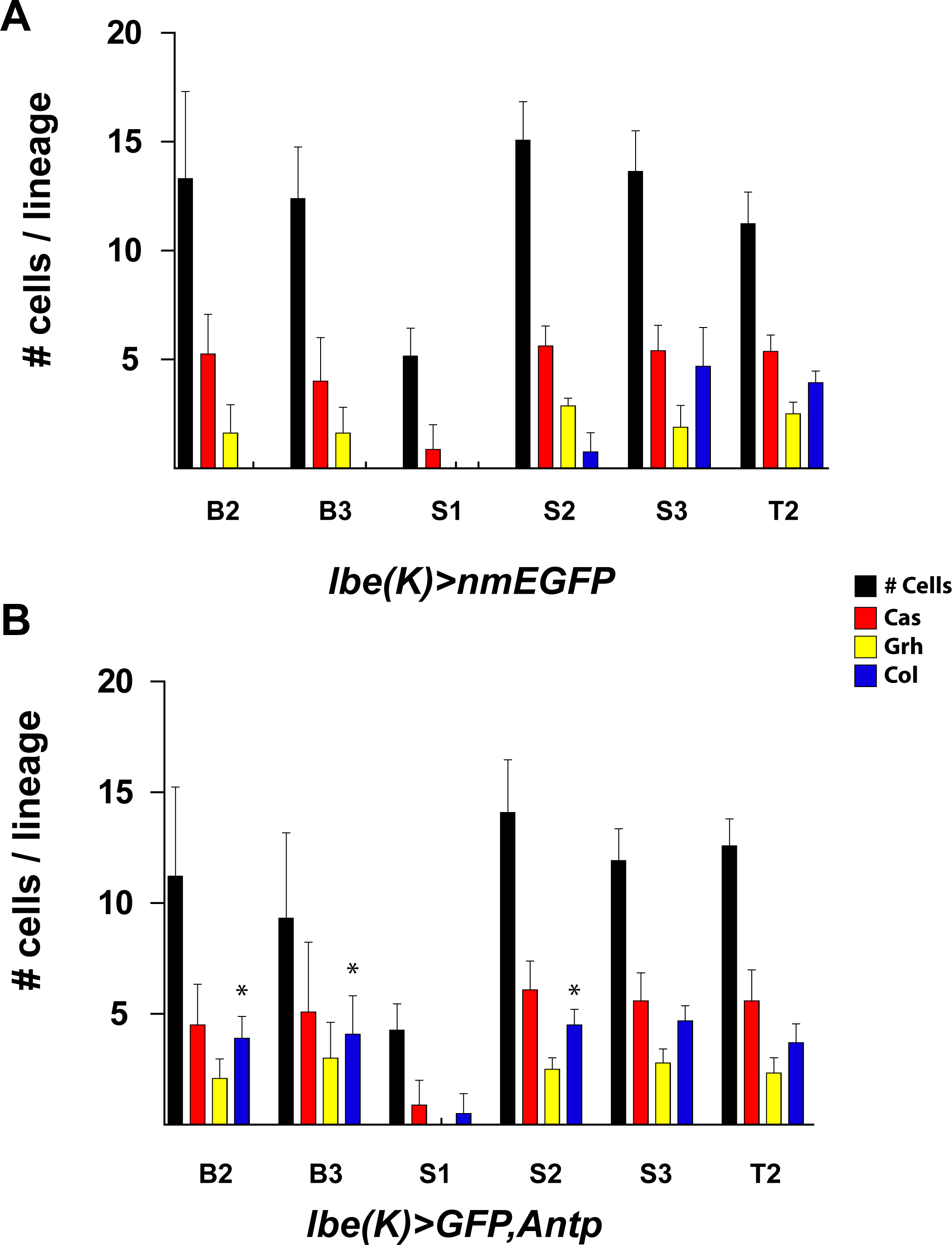

Supplement: Figure S10 — Antp misexpression does not lead to homeotic transformation of anterior NB 5–6 lineages. Quantification of the number of cells expressing GFP, Cas, Grh, and Col in the anterior NB 5–6 lineages, in control (top) and Antp misexpression (bottom), at stage 15 (cells/lineage; n>12 lineages). Whereas Col is ectopically activated by Antp, there are no significant changes in NB 5–6 lineage cell numbers, nor in Cas or Grh cell numbers. Asterisks denotes significant difference compared to control (p<0.01; Student two-tailed test). Genotypes: (A) lbe(K)-Gal4, UAS-nmEGFP/+, (B) lbe(K)-Gal4, UAS-nmEGFP/UAS-Antp. (0.21 MB TIF) [file pbio.1000368.s010.tif]
